# Using spectral decomposition of the signals from laurdan-derived probes to evaluate the physical state of membranes in live cells

**DOI:** 10.12688/f1000research.11577.2

**Published:** 2017-08-01

**Authors:** Serge Mazeres, Farzad Fereidouni, Etienne Joly

**Affiliations:** 1 Membrane and DNA Dynamics Team, Institut de Pharmacologie et de Biologie Structurale, CNRS, Université de Toulouse, Toulouse, F-31077, France; 2 Department of Pathology and Laboratory Medicine, University of California Davis Medical Center, CA 95817, CA, 4400, USA

**Keywords:** Membrane, lipid bilayer, microdomains, solvatochromic, spectral unmixing, spectral decomposition, rafts, biphoton microscope.

## Abstract

*Background: *We wanted to investigate the physical state of biological membranes in live cells under the most physiological conditions possible.

*Methods:* For this we have been using laurdan, C-laurdan or M-laurdan to label a variety of cells, and a biphoton microscope equipped with both a thermostatic chamber and a spectral analyser. We also used a flow cytometer to quantify the 450/530 nm ratio of fluorescence emissions by whole cells.

*Results: *We find that using all the information provided by spectral analysis to perform spectral decomposition dramatically improves the imaging resolution compared to using just two channels, as commonly used to calculate generalized polarisation (GP). Coupled to a new plugin called Fraction Mapper, developed to represent the fraction of light intensity in the first component in a stack of two images, we obtain very clear pictures of both the intra-cellular distribution of the probes, and the polarity of the cellular environments where the lipid probes are localised.

Our results lead us to conclude that, in live cells kept at 37°C, laurdan, and M-laurdan to a lesser extent, have a strong tendency to accumulate in the very apolar environment of intra-cytoplasmic lipid droplets, but label the plasma membrane (PM) of mammalian cells ineffectively. On the other hand, C-laurdan labels the PM very quickly and effectively, and does not detectably accumulate in lipid droplets.

*Conclusions:* From using these probes on a variety of mammalian cell lines, as well as on cells from *Drosophila* and *Dictyostelium discoideum*, we conclude that, apart from the lipid droplets, which are very apolar, probes in intracellular membranes reveal a relatively polar and hydrated environment, suggesting a very marked dominance of liquid disordered states. PMs, on the other hand, are much more apolar, suggesting a strong dominance of liquid ordered state, which fits with their high sterol contents.

## Introduction

The lipid bilayer is the main architectural component of biological membranes, in which a variety of proteins are more or less deeply embedded. Over the past two decades, it has become widely accepted that biological membranes are not simply homogeneous seas of lipids studded with proteins, but contain microdomains that play crucial roles in the assembly of signalling platforms and in intracellular transport between various compartments ( Lingwood & Simons, 2010). Although the physical forces that govern membrane microdomains are still poorly understood, it is now commonly accepted that the degree of order in the lipids is highly relevant to the formation of lipid domains in eukaryotic cell membranes ( Rosetti *et al.*, 2017; Sezgin *et al.*, 2017).

Two important characteristics of membrane microdomains are their small size (typically below 100 nm, i.e. too small to be seen by standard optical microscopy) and their very dynamic nature ( Ali *et al.*, 2006). Because of these characteristics, the physical state of biological membranes, and of the microdomains within them, has proven very difficult to observe and characterise. One way to circumvent these difficulties is to use fluorescent lipids with solvatochromic properties, i.e. fluorescent probes whose emission spectra are influenced by the polarity of their environment ( Klymchenko & Kreder, 2014). Because microdomains are commonly viewed as structures with greater lipid order than the surrounding membrane lipids, and because this greater order is linked to a reduced exposure to water and thus to a less polar environment, the emission spectra of solvatochromic lipid probes differ when they are within or outside membrane microdomains.

The probes most frequently used for this type of approach belong to five main families: laurdan-, Nile Red- (NR12S), ANEPPDHQ-, 3-hydroxyflavone- and pyrene-based lipid probes ( Klymchenko & Kreder, 2014; Le Guyader *et al.*, 2007; Niko *et al.*, 2016; Owen *et al.*, 2011). Among these, we prefer the probes derived from laurdan, for two main reasons. Firstly, laurdan and its derivatives have no marked preference for ordered or disordered lipid environments and thus tend to distribute evenly in lipid bilayers harbouring co-existence of lipid domains in different physical states. Secondly, the emission spectra of the laurdan-family probes show a relatively clear-cut dichotomy in their emission spectra in different types of membranes: in the apolar environments of phospholipid bilayers in either liquid ordered (Lo) or solid (So) states their emission maxima are at 440 nm, whereas when they are in the relatively polar environments of membranes in the liquid disordered state (Ld) their emission maxima are at 490 nm ( Bagatolli *et al.*, 2003; Parasassi & Gratton, 1995).

The most widely accepted model to explain the ‘red shift’ of laurdan from an emission maximum of 440 nm to a maximum of 490 nm in more polar environments is based on the reorientation, or relaxation, of structured water molecules in the direct vicinity of the probe ( Bagatolli, 2013). Such a model explains the ‘all or nothing’ response in the emission spectrum of the laurdan-family probes: in the excited state, which has a half-life of a few nanoseconds, similar to the timeframe of water relaxation, either the fluorochrome does not dissipate energy towards a water molecule and the emitted photon has a wavelength around 440 nm, or there is a water molecule in the direct vicinity of the fluorochrome towards which relaxation of part of the excitation energy can be transferred, and the emitted photon will then have a wavelength around 490 nm.

The ANEPPDHQ, Nile Red and pyrene-derived probes, by contrast, undergo more progressive shifts in their emission maxima depending on the dielectric constants of their environments ( Jin *et al.*, 2006; Sezgin *et al.*, 2014). Furthermore, the shifts in emission spectra of these probes are not necessarily linked to the degree of order of the bilayers ( Amaro *et al.*, 2017). Indeed, when the ANEPPDHQ or Nile Red probes are inserted in model bilayers of pure phospholipids below their transition temperature, i.e. bilayers in a highly ordered So or gel state, their emission spectra tend to resemble those corresponding to a Ld state. This may be explained if these probes tend to be excluded from the crystalline mesh that the phospholipids form when they switch to a gel or So state, and thus they may accumulate in imperfections or 'cracks' in the bilayer where they will be more exposed to water. A similar phenomenon of red-shifted emission occurs with laurdan when it is inserted into model bilayers of pure glycosphingolipids, especially those bearing large headgroups; this may be explained by the formation of sphingolipid aggregates resulting in the exposure of the probe to water molecules ( Bagatolli *et al.*, 1998).

Laurdan was first synthesised by Weber in 1979 ( Macgregor & Weber, 1981; Weber & Farris, 1979); it has since been widely used to investigate the physical state of biological membranes ( Gaus *et al.*, 2005; Jay & Hamilton, 2017; Yu *et al.*, 1996). For a thorough review of laurdan’s history and properties, the reader is referred to the book chapter by Luis Bagatolli ( Bagatolli, 2013). Previous studies, however, were mostly of cells at room temperature that were very often fixed with a crosslinking agent such as formaldehyde. We wanted to study the plasma membranes (PMs) of living cells at 37°C exposed to as little disturbance as possible. At 37°C, however, laurdan tends to accumulate in intracellular compartments and to label the PM rather poorly. C-laurdan, a probe first synthesised and characterised in 2007 was derived from laurdan by adding a carboxyl group to the polar head, and labels the PM of eukaryotic cells more effectively than laurdan ( Kim *et al.*, 2007). In 2014, we reported an optimised protocol for the synthesis of C-laurdan and of M-laurdan, an intermediate in the synthesis that showed promising properties ( Mazeres *et al.*, 2014).

Here, we have used these three laurdan family probes to label live cells maintained in tissue culture medium and at 37°C for the duration of the labelling procedure and observation. This, combined with an analysis based on spectral decomposition, has allowed us to obtain a clearer picture of the physical state of membranes in live cells compared with previous studies based on generalised polarisation (GP), which is calculated simply from the fluorescence emissions at 440 and 490 nm. Our study provides an estimate of the proportion of apolar *versus* polar environments in the lipid bilayers of various cellular compartments.

## Methods

### Chemicals and probes

Laurdan, M-laurdan and C-laurdan were all synthesised in-house as previously described ( Mazeres *et al.*, 2014). Aliquots of 2 mM stock solutions in DMSO were kept in long-term storage at -20°C; when needed for experiments, aliquots were kept at 4°C for, at most, six weeks. Various staining procedures were used for different experiments, as described later. Nile Red was obtained from Sigma; staining was performed at 10ng/ml for 30–60 min at 37°C in tissue culture medium without serum. LysoTracker Red DND-99 was obtained from Invitrogen; staining was performed at 20 ng/ml (50 nM) for 30–60 min at 37°C in tissue culture medium without serum. Methyl-β-cyclodextrin (MßCD) (cell culture tested) was obtained from Sigma.

### Multilamellar large vesicles

Multilamellar large vesicle (MLV)s were prepared as follows. DPPC (1,2-dipalmitoyl-sn-glycero-3-phosphocholine), DOPC (1,2-dioleoyl-sn-glycero-3-phosphocholine) and cholesterol were purchased from Sigma–Aldrich, and prepared as stock solutions in chloroform. The appropriate amounts of these stock solutions to obtain a final concentration of either 100 μM DOPC (for Ld MLVs), or 60 μM DPPC and 40 μM cholesterol (for Lo MLVs) in a final volume of 3 ml were placed in glass tubes. The chloroform solvent was first evaporated under nitrogen flow and then under vacuum for 2 hours. Three ml of MOPS buffer (3-(N-morpholino)propane sulfonic acid, 10 mM, pH 7.3, NaCl 100 mM, EDTA 10 μM) was then added either at room temperature (for the DOPC MLVs) or at 50°C (for the MLVs containing 60% DPPC and 40% cholesterol). The tubes were then vortexed vigorously for 30 seconds to form MLVs. Stock solutions of the various probes were prepared in DMSO such that 1/1000 v/v of the probe solution was added to the aqueous suspension of MLVs. To prevent dye aggregation in water, the probes were always added while vortexing the tubes containing the MLVs. They were then incubated at room temperature for at least one hour before analysis in a QM4 Spectrofluorimeter (Photon Technology International), or observation by biphoton microscopy (see below).

### Cells

Mammalian cells were all grown in DMEM with 10% fetal calf serum (FCS) at 37°C with 5% CO _2_ and passaged by trypsinisation every three or four days. Hela, HCT116 and L(tk-) cells were obtained from the ATCC. The primary culture of human foreskin fibroblasts was a gift from Laure Gibot (IPBS, Toulouse, France). *Drosophila melanogaster* Kc cells were provided by Vanessa Gobert (CBD, Toulouse, France) and were grown at room temperature in Shields and Sang medium with 10% FCS. *Dictyostelium discoideum* AX2 cells, provided by François Letourneur (DIMNP, Montpellier, France), were grown in HL5 medium at room temperature.

### Cell preparation and staining

All mammalian cells were grown for 2–4 days on glass coverslips in six-well plates, with 2 ml of medium in each well. On the day of the assay, the glass coverslips were placed inside stainless steel culture chambers (either from SKE Research Equipment (Milan) or from ThermoFisher), which had been pre-warmed in the same incubator as the cells for at least 30 minutes. One ml of the medium that the cells were growing in was then transferred from the well of the six-well plate to the chamber, and the chamber containing the coverslip with the cells on it was then returned to the incubator for at least 30 minutes. The medium was then rinsed once with 1ml of DMEM with neither serum nor phenol red, which had been pre-warmed to 37°C. The probes (in 1µl DMSO) were placed in an Eppendorf tube, and 1 ml of pre-warmed DMEM was quickly pipetted up and down twice in the tube before transferring onto the cells. The chamber was then returned to the tissue culture incubator for various times (at least 20 minutes for C-laurdan, and 45 minutes for M-laurdan and laurdan) before observation. For the time-courses, such as the ones shown in Figure 7, C-laurdan was added by pipetting 500 µl out of the chamber, pipetting this liquid up and down twice into an Eppendorf tube containing the adjusted amount of probe in DMSO, and returning the 500 µl to the observation chamber with pipetting up and down a few times.

*Drosophila* Kc cells were grown for 48 hours on coverslips coated with poly-L-lysine (0.1 mg/ml for 1 hour followed by three rinses in PBS), and stained with 800 nM C-laurdan in Shields and Sang serum-free medium. *Dictyostelium* AX2 cells, were incubated for ~two hours in HL5 medium at room temperature to allow them to adhere to untreated coverslips. They were then stained with 800 nM C-laurdan in Sörensen's buffer.

Since the probes fluoresce only in a lipid environment, the cells were imaged in the staining medium. (Note that if rinsing is necessary, it should be done with medium without serum, or the labelling will fade rapidly, especially in the case of C-laurdan. If cells need to be returned to serum-containing medium, for example for very long incubation times, one should consider using laurdan or M-laurdan.)

### Spectral imaging by biphoton microscopy

Mammalian cells were maintained at 37°C, 5% CO _2_ throughout the imaging procedure. All images were recorded on a LSM 710 NLO-Meta confocal laser-scanning microscope controlled by Zen software (2010B SP1, v 6.0.0.485), equipped with a 40x/1.2 water immersion objective, a gas-controlled thermostatic chamber and a spectral detection module (Zeiss, Germany), and coupled to a biphoton laser source (3 watts at 800 nm; Chameleon Vision II, Coherent, France). When tuned to 720 nm, the laser delivered 2.14 watts, and with the AOTF set to 4%, the average measured power delivered at the level of the objective was 6 mW.

Unless otherwise specified, the settings of the microscope were: biphoton tunable laser set at 720 nm; 2–4% power; pixel dwell time of 6.3 µsec; average on four measures; pinhole open (600) or sometimes closed to 150 (never more) to gain higher resolution of close-up pictures (zoom 4 and above). When the settings differed from these, the specific details are given in the legends of the corresponding figures. The images were all acquired as stacks in lambda-mode with spectral resolution steps of 9.8 nm. For acquisitions with the laurdan-derived probes, we recorded the 17 channels between 418 nm and 584 nm plus a channel for transmitted light. When Nile Red or LysoTracker Red were included in the experiment, we recorded the 29 channels between 418 nm and 700 nm, plus a channel for transmitted light. All the images shown in all the figures are representative of at least six images (of six different fields), acquired on at least two different days.

### Image analysis

Although the Zen software we used for acquisition of images on the Zeiss microscope allows extensive and convenient image analysis, including spectral decomposition (called ‘linear unmixing’ in the Zen software), this commercial software is not widely available. We have therefore chosen to base all the image analyses reported in this paper on ImageJ software (current version 1.48V, Java 1.6.0_65) complemented with various plugins, which are all open code software and freely available.

The PoissonNMF plugin ( https://github.com/neherlab/PoissonNMF) performs nonnegative matrix factorisation to allow spectral decomposition without knowing the initial spectra ( Neher *et al.*, 2009). Here, we used it to perform spectral decomposition analysis of reference spectra acquired separately on MLVs of defined composition. The ‘blind’ mode used on live cells labelled with laurdan-derived probes usually gave results very similar to those obtained by using reference spectra, but the spectral identification was sometimes less accurate, especially when the signal to noise ratios were low.

When using fixed spectra with the PoissonNMF plugin, we used ten iterations (as recommended by Richard Neher, personal communication). The other values were the default settings suggested by the program: subsamples, 2; segregation bias, 0; saturation threshold, 4000; background threshold, 50; background spectrum, minimal values, and the spectral values were specified manually to match those of the spectral analyser of the biphoton microscope. Note: for the PoissonNMF plugin to function correctly when using specified spectral values, it is essential that the number of channels specified matches exactly the number of channels in the stack. In the process of assembling practical examples of the procedure with step by step instructions for an updated version of our manuscript, we came to realize that this pitfall can be avoided by simply relying on the default spectral values of PoissonNMF (min 480nm, max 560nm). The allocated wavelengths will then be erroneous, but this will have absolutely no influence on the unmixing results. For more information, see the step by step instructions provided as Supplementary material.

The Fraction Mapper plugin (see Software and data availability) was developed specifically for this study. This plugin converts the two-image stack obtained through spectral decomposition into fractional intensities by dividing the unmixed images by the total intensity image. The summation of fractional intensities is always 1 and since we only unmix two components, the fractional intensity can be indexed with only one number. Next, the fractional intensity is mapped on to the total intensity image and it is colour-coded using either readily available or custom made look-up table (LUT)s. The saturation of the colours is scaled with the pixel value of the total intensity. The plugin allows the choice of various LUTs and also thresholding of the image. After running the plugin on the stack of unmixed images, the plugin asks for the desired LUT, it generates the colour-coded fractional intensity image and also a 2D histogram, where the frequency is colour-coded. The axes of the histogram plot are the intensity of the two unmixed images. The plugin allows the user to make regions of interest on the 2D plot and to project them back into the fractional colour-coded image, rather like the gating of flow cytometry plots.

### Flow cytometry

For analysis by flow cytometry, Hela cells were harvested by trypsinisation, and a single-cell suspension was prepared in tissue culture medium (DMEM with 10% FCS). Cells were then washed twice in pre-warmed DMEM with neither serum nor phenol red before adding the probes (1/1000 v/v of the DMSO stocks) followed by incubation in a 37°C water bath placed near the flow cytometer.

For cholesterol depletion, a fresh 10X stock solution of MßCD was prepared at 50mM (66mg/ml of MßCD powder) in PBS on the day of each experiment. Cell suspensions were rinsed twice in DMEM without serum before incubating with 5mM MßCD for 30 minutes at 37°C. Cells were then rinsed twice with warm DMEM without serum before staining with the laurdan-family probes. (Note, treatment of the cells with MßCD after labelling with the laurdan-family probes results in very effective removal of the probes.)

At the end of the incubations, the cells were analysed on a Beckton Dickinson LSR II flow cytometer equipped with a 355 nm UV laser, and the following set of filters were used for the analysis of the emitted light: first channel: 505LP + 530/30 BP; second channel 450/50 BP. For each sample, 5000 cells gated on FSC/SSC were analysed.

## Results

### Using spectral decomposition to study colocalisation of two stains in live cells shows that laurdan and M-laurdan accumulate in lipid droplets

In a previous study, we compared the photophysical properties of C-laurdan and two intermediates in its synthesis, M-laurdan and MoC-laurdan, to those of laurdan ( Mazeres *et al.*, 2014). We found that the probes distributed very differently inside live cells. Laurdan stained predominantly cytoplasmic ‘vesicles’, which we speculated might belong to the endolysosome pathway ( Mazeres *et al.*, 2014). Since then, we observed that these vesicular bodies were very apolar, since they were seen much better at 440 nm than at 490 nm ( Supplementary File 1), which suggested that they may in fact be lipid droplets. To discriminate between these two possibilities, we wanted to perform double labelling with either LysoTracker Red (which labels lysosomes) or Nile Red (which labels lipid droplets) and one of each of the three laurdan-derived probes. We found, however, that these vesicles were also very mobile ( Supplementary File 1), so we could not perform successive image acquisitions to investigate whether the laurdan-based probes co-localized with LysoTracker Red or with Nile Red.

**Figure 1.**
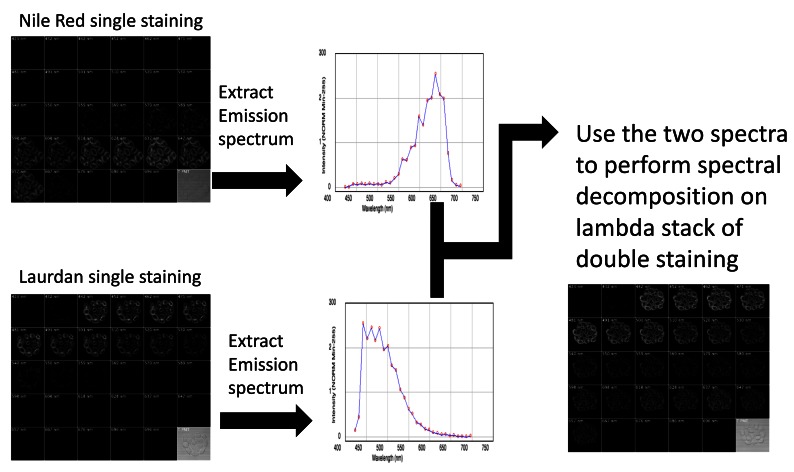
Scheme of the double staining and spectral decomposition procedure. To perform spectral decomposition on live cells labelled with two fluorescent dyes, we first acquired lambda stacks of cells labelled with either Nile Red or laurdan. The emission spectra of the dyes were then extracted from those stacks. Those emission spectra were then used to perform spectral decomposition on the lambda stacks acquired from cells stained with both dyes (bottom right panel).

**Figure 2.**
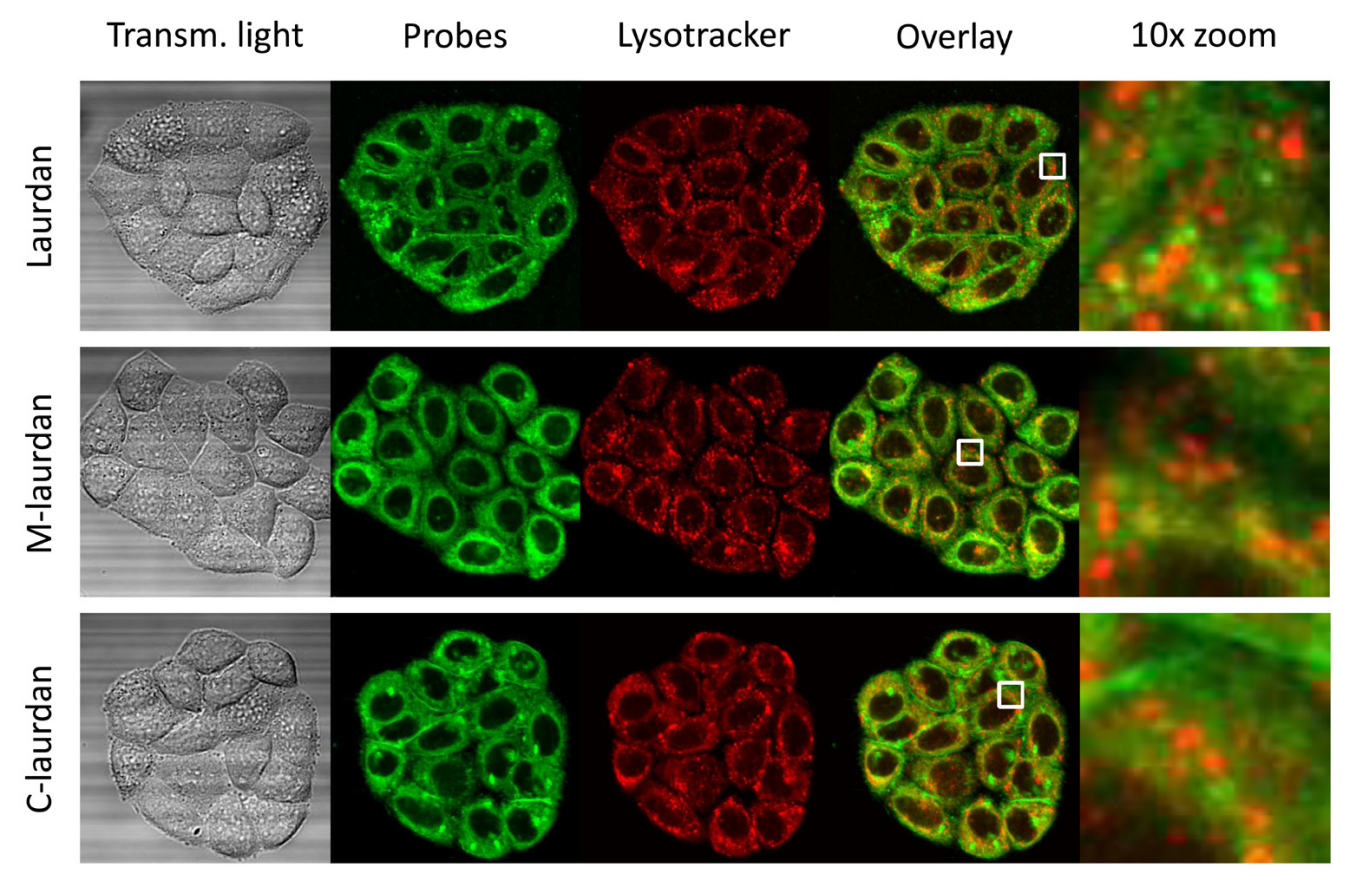
The intra-cellular vesicles labelled by Laurdan, and to a lesser extent by M-laurdan, do not co-localize with lysotracker. Hela cells were grown on glass coverslips for four days before double-labelling with lysotracker red and either Laurdan (first line), M-laurdan (second line) or C-laurdan (third line), as described in M&M. The live cells, kept at 37°C, were then imaged over 30 channels: 29 fluorescence channels spanning from 420 to 700 nm, plus transmitted light (First column). The stacks were then used to perform spectral deconvolution with the poisson NMF pluggin, using the spectra acquired with cells labelled with only one probe as references. The second column shows the signals allocated to the Laurdan-family probes, artificially coloured in green. The third column shows the signals allocated to lysotracker red, artificially coloured in red. The fourth column shows an overlay of the two signals, and the fifth column shows 10× zoom corresponding to the white squares in the fourth column. Width of the squares: 10 µm.

**Figure 3.**
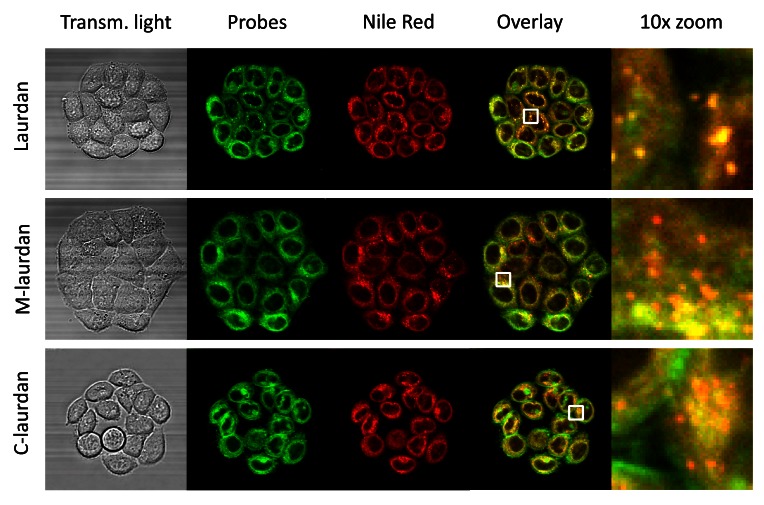
The intra-cellular vesicles labelled by Laurdan co-localize perfectly with Nile Red labelling, which labels lipid droplets. Hela cells were grown on glass coverslips for four days before double-labelling with Nile red and either Laurdan (first line), M-laurdan (second line) or C-laurdan (third line), as described in M&M. The live cells, kept at 37°C, were then imaged over 30 channels: 29 fluorescence channels spanning from 420 to 700 nm, plus transmitted light (First column). The stacks were then used to perform spectral deconvolution with the poisson NMF pluggin, using the spectra acquired with cells labelled with only one probe as references. The second column: shows the signals allocated to the Laurdan-family probes, artificially coloured in green. The third column shows the signals allocated to Nile red, artificially coloured in red. The fourth column shows an overlay of the two signals, and the fifth column shows 10× zooms corresponding to the white squares in the fourth column. Width of the squares: 10 µm.

To circumvent this problem, we used the lambda mode of acquisition on our microscope to collect the fluorescent light emitted by the two dyes at exactly the same time, and then ‘unmixed’ the signals of the two probes by spectral decomposition. Thus, the mobility of the vesicles in live cells no longer confounded the interpretation of localisation of the two dyes. Although the bi-photon excitation at 720 nm was clearly suboptimal for those two red probes, it still worked well enough for the probes to be detected. For Nile Red, we actually found that we had to dilute the probe tenfold compared to what is usually reported in the literature ( Greenspan *et al.*, 1985) to obtain intensities of signals comparable with those of the laurdan-based probes (see methods). To unmix the emission spectra, we first used cells labelled with only one probe to acquire the corresponding emission spectrum from 420 to 700 nm. We then used these emission spectra to perform spectral decomposition on images of cells stained with two probes ( Figure 1). Decomposition revealed that the staining patterns obtained with LysoTracker Red did not coincide with those obtained with any of the three laurdan probes ( Figure 2). By contrast, the staining patterns obtained with Nile Red were essentially superimposable on those obtained with all three laurdan-derived probes, and in particular with laurdan: there was an almost perfect coincidence of the staining patterns obtained with laurdan and Nile Red ( Figure 3). We conclude from this that the cytoplasmic vesicles detected with laurdan (and to a certain extent with M-laurdan) do not belong to the endolysosome pathway, as we initially postulated, but are lipid droplets. These findings are supported by those of Owen and colleagues who noted in the troubleshooting table of their article ( Owen *et al.*, 2012a) that lipid droplets tend to sequester laurdan. As previously documented by time-resolved microscopy ( Ghosh *et al.*, 2013), the environment inside lipid droplets is relatively viscous and very apolar, but not necessarily very ordered. Our findings are, therefore, a useful reminder that the solvatochromism of the laurdan family probes reflects not the order but the polarity of their environment; in other words, the presence or absence of water molecules in the immediate environment of the probes.

Of note, in the clumps of 15–20 cells labelled with either laurdan or M-laurdan ( Figure 2 and Figure 3), the labelling intensity of the cells inside the clumps appeared somewhat weaker than the labelling of cells at the periphery. This may be due to inaccessibility of the probes to the cells inside the clumps, but more likely reflects a difference in the lipid composition of the cells in the crowded environment inside the clumps when compared to the cells at the periphery, as suggested previously ( Frechin *et al.*, 2015; Gray *et al.*, 2015).

### Using spectral decomposition on signals from solvatochromic probes

**Figure 4.**
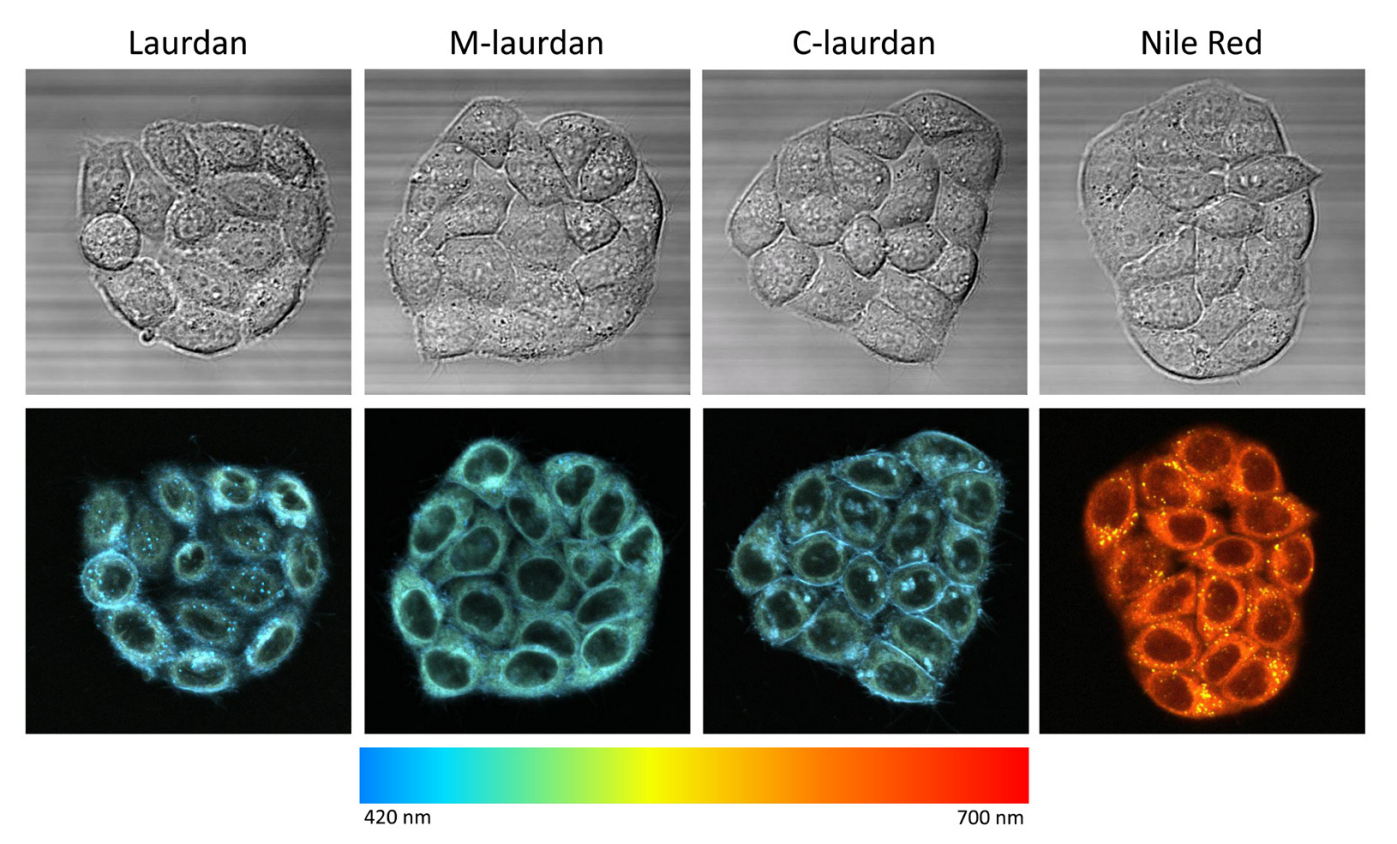
Colour-coded representation of lambda stacks reveals different colours for different cellular compartments, suggesting that it may be possible to perform spectral deconvolution. Colour coding was achieved using the built-in ‘time-lapse colour coding’ pluggin of ImageJ, after converting the 29 channels stacks into 29 frames stacks. In designing this lut (called rainbeau), we attempted roughly to match the wavelengths of the corresponding channels.

To reconstitute colour pictures from the stacks of images obtained in lambda mode, we used a chromatic LUT designed to match the wavelengths of the corresponding channels ( Figure 4). For each of the four solvatochromic probes, we noticed differences in the colours emitted by various cellular compartments. For the laurdan-based probes, as expected from the results of several previous studies ( Golfetto *et al.*, 2013; Niko *et al.*, 2016; Owen & Gaus, 2010; Owen *et al.*, 2011; Yu *et al.*, 1996), the cytoplasm was slightly greener than the blue PMs and lipid droplets. With Nile Red, however, there was a striking difference between the staining of the lipid droplets, which were bright yellow, and the staining of the intracellular membranes, which appeared red–orange. This solvatochromism of Nile Red in lipid droplets, which is due to their very hydrophobic nature, was documented previously ( Greenspan & Fowler, 1985). 

These observations led us to wonder whether we could decompose the various spectral components of the different colours emitted by the laurdan-derived solvatochromatic probes by a similar approach to that which we used to unmix the signals from two probes above. Indeed, rather than a progressive shift in their emission maximum, the laurdan-derived probes have more of a bimodal response to changes in the dielectric constants of their environment, which is particularly well marked between the more water-exposed environment of the probes in lipid membranes in disordered phase and the more hydrophobic, or apolar, environment of the probes in more ordered membranes, whether Lo or gel phases ( Bagatolli, 2013; Bagatolli *et al.*, 2003; Parasassi & Gratton, 1995). Thus, these bimodal fluorescence signals should be particularly amenable to spectral decomposition, and this might allow us to evaluate the proportion of ordered and disordered states of cell membranes.

**Figure 5.**
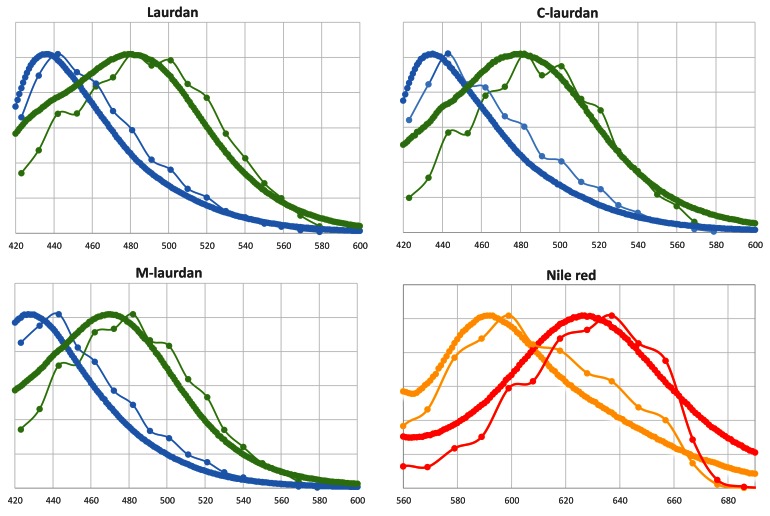
Emission curves obtained with the biphoton microscope are very similar to those obtained on a standard spectrofluorimeter. The four fluorescent lipid probes were inserted into either Lo or Ld MLVs and their emission curves were recorded in cuvettes on a spectrofluorimeter (thick lines), or imaged on the biphoton microscope in the same conditions as used for live cells. After defining appropriate ROIs in the lambda stacks, the emission curves of individual MLVs were extracted using the ImageJ software. The thin lines correspond to the average curves obtained from 6–10 separate MLVs (the error bars are smaller than the symbols). Lo, DPPC–chol MLVs (blue or orange); Ld, DOPC MLVs (green or red).

We first compared the emission spectra obtained from fluorescently labelled DPPC–cholesterol (Lo) or DOPC (Ld) MLVs in a regular spectrofluorimeter to those obtained with the biphoton microscope in lambda mode from 480 to 600 nm for the three laurdan-based probes, and from 520 to 700 nm for Nile Red ( Figure 5). The emission curves extracted using ImageJ software on the MLV images acquired on the biphoton microscope were remarkably similar to those obtained from the same MLVs in cuvettes in the spectrofluorimeter. Thus, the spectral analyser of the biphoton microscope can be used to analyse emission spectra in two dimensions at the submicrometer scale.

**Figure 6.**
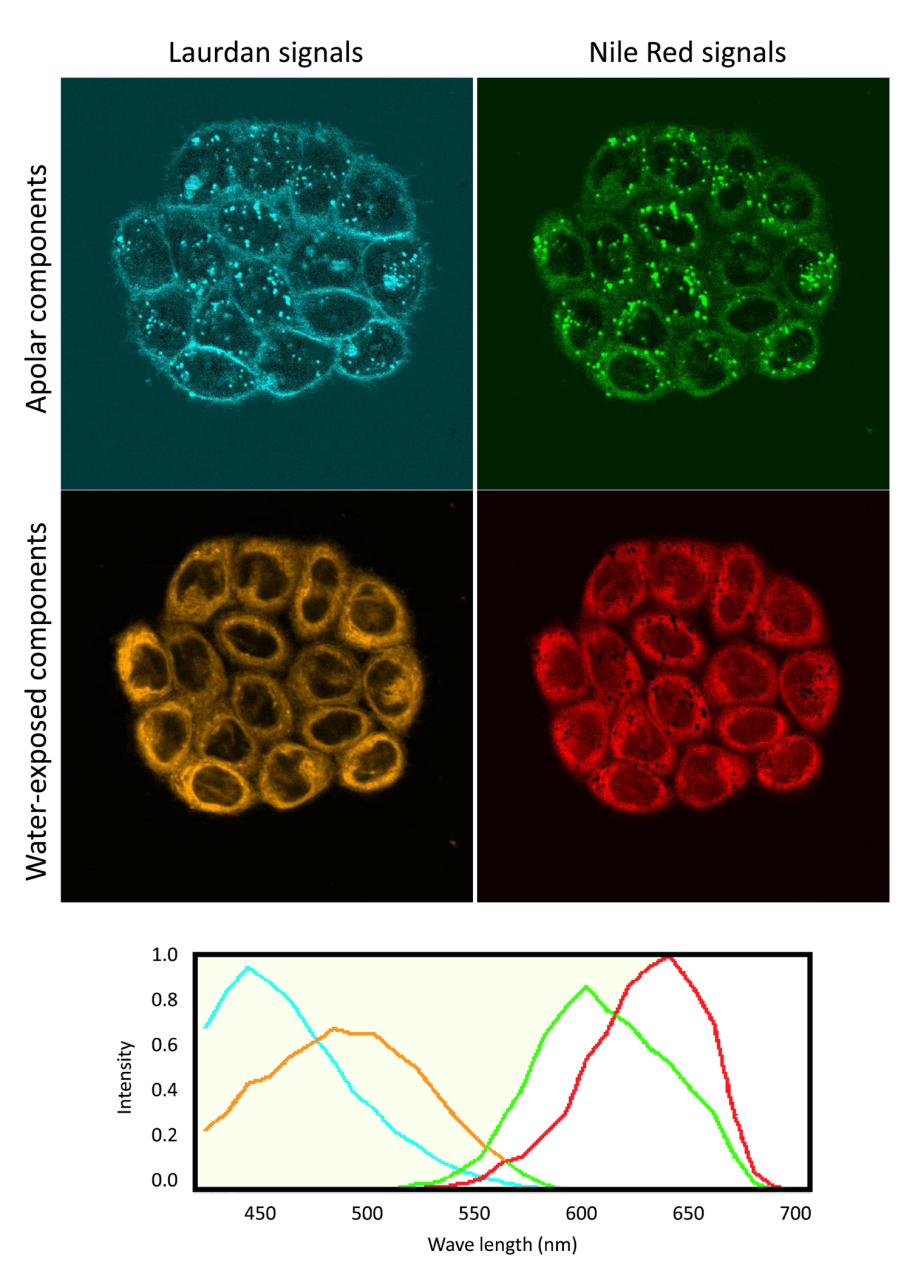
Using the spectra acquired on MLVs and the poisson NMF pluggin in ImageJ, the acquired image stacks can be efficiently separated in as many as 4 different colours. The poisson-NMF pluggin was used to perform spectral deconvolution on the lambda-stack obtained with live cells labelled with both Laurdan and Nile red (see Figure 3). Reference curves used (lower panel) were those obtained on MLVs labelled either with Laurdan (DPPC-cholesterol (Lo): cyan curve, DOPC(Ld): orange curve) or with Nile red (DPPC-cholesterol (Lo): green curve, DOPC(Ld): red curve). Settings used were all as described in M&M. The four panels correspond to: Upper-left (cyan): Laurdan in apolar (hydrophobic) environment; Lower-left (orange): Laurdan in water-exposed environment; Upper-right (green): Nile red in apolar (hydrophobic) environment; Lower-right (red): Nile red in water-exposed environment. Laurdan Nile Red 02 from 07 April 2015.

We then tried our decomposition approach on the most challenging task available to us, i.e. we performed spectral decomposition into four different channels on stacks of 29 images acquired after double staining with laurdan and Nile Red ( Figure 6). By using the four emission curves obtained with each probe on either DPPC–cholesterol (Lo) or DOPC (Ld) MLVs to perform spectral decomposition with the PoissonNMF plugin, we obtained a very convincing separation of the signals, where laurdan was found to be in an apolar, water-poor environment in lipid droplets and at the PM, whilst being much more exposed to water in the intra-cellular membranes. For Nile Red, there was effectively no detectable staining of the PM, but a marked dichotomy between hydrophobic lipid droplets and more hydrophilic intra-cellular membranes.

From these results, we came to suspect that spectral decomposition could turn out to be a very powerful approach to analyse the cellular distribution of solvatochromic probes such as laurdan, and more importantly, the actual physical state of their environment in different cellular compartments.

### Improving the reproducibility of the results obtained with C-laurdan

Most signalling events take place at the PM. Given its cellular distribution, C-laurdan appeared as the best suited probe to investigate such events. At the beginning of our study, however, we found that the intra-cellular distribution of C-laurdan, as well as the polar/apolar ratios in various cellular compartments, were very variable, not only from one experiment to another, but even between cells on the same coverslip.

The first factor that we identified as having a major influence on the heterogeneity of our results was cellular stress. This could result, for instance, from increased osmotic pressure due to water evaporation when we carried out the staining steps in small volumes, from sudden temperature changes if we used buffers that had not been pre-warmed to the same temperature as that of the cells, or simply from unstable, oscillating temperatures when we used chambers of insufficient thermal inertia.

Intra-sample homogeneity was much improved by the use of heavy stainless steel chambers in which we could perform the staining steps with large volumes (0.5 – 1 ml) of buffers pre-warmed to 37°C (see Methods).

**Figure 7.**
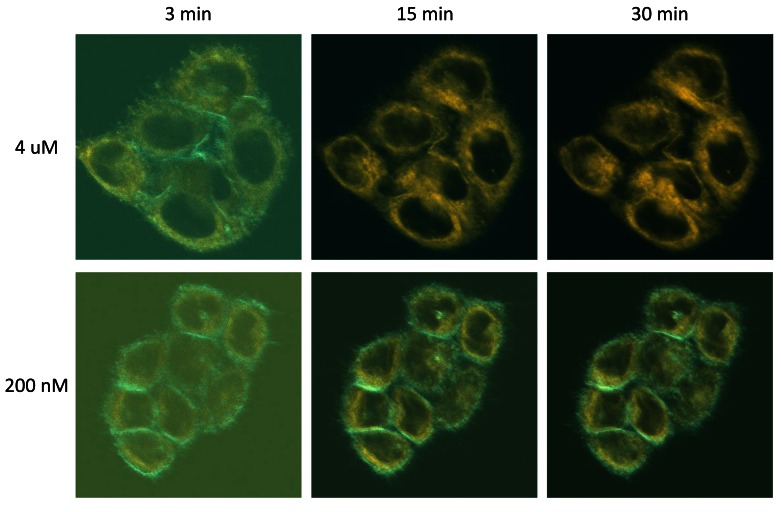
High concentrations of C-laurdan can result in autoquenching at the plasma membrane. Hela cells growing on coverslips were placed in observation chambers in 1 ml of serum-free medium and placed in the thermostatic chamber of the biphoton microscope. After focusing on a chosen group of cells, C-laurdan was added to a final concentration of either 4 µM (top panels) or 200 nM (bottom panels). Lambda stack images were then taken at the indicated times, with the laser power set at 2% for staining with 4 µM, and 4% for 200 nM C-laurdan. All other settings were as described in Methods and spectral decomposition was carried out as described in Methods. The pictures shown are dual-colour overlays. Cyan, C-laurdan in apolar environments; orange, C-laurdan in water-exposed environments. The duller aspect of the pictures taken at 3 minutes is due to the fact that, at this early time point, the probe had not had time to stain the cells fully, resulting in low signal to noise ratios.

Despite these improvements, we were still witnessing very significant variability in the staining patterns of the cells stained with C-laurdan. In particular, we noticed that these patterns tended to change over time, with the high prominence of the apolar component at the PM that we obtained at the very early time points (< 5 min) tending to fade, and even disappear almost completely over time (cyan colour in Figure 7 upper row). We found out, however, that this did not occur when we used lower concentrations of the probe (see lower panel of Figure 7).

Having observed this, we felt that it was important to document at what precise concentrations this phenomenon was occurring. In addition, we also wanted to optimise our staining protocols with all three probes for both the concentrations used and the incubation times. One of the major drawbacks of fluorescence microscopy, however, lies with the difficulty in generating quantitative measurements. Another difficulty of microscopy lies with the relatively small number of samples that can be analysed.

### Using flow cytometry for quantitative analyses

To circumvent this problem, we turned to flow cytometry, which allows quantitative analysis of many cells, in numerous different samples to be performed. For each cell, emissions in the 400–500 and 500–560 nm windows were recorded simultaneously. We realised that the ratio of the signals recorded in the first channel over that in the second should vary with the proportion of the probe emitting fluorescence in apolar *versus* water-containing environments, similarly to the GP factor, which is commonly used for the analysis of laurdan-based studies. Given that the intensities of the signals are completely dependent of the instrument settings, however, the values obtained are purely relative, and can thus only be used to compare the samples of one experiment with one another.

**Figure 8.**
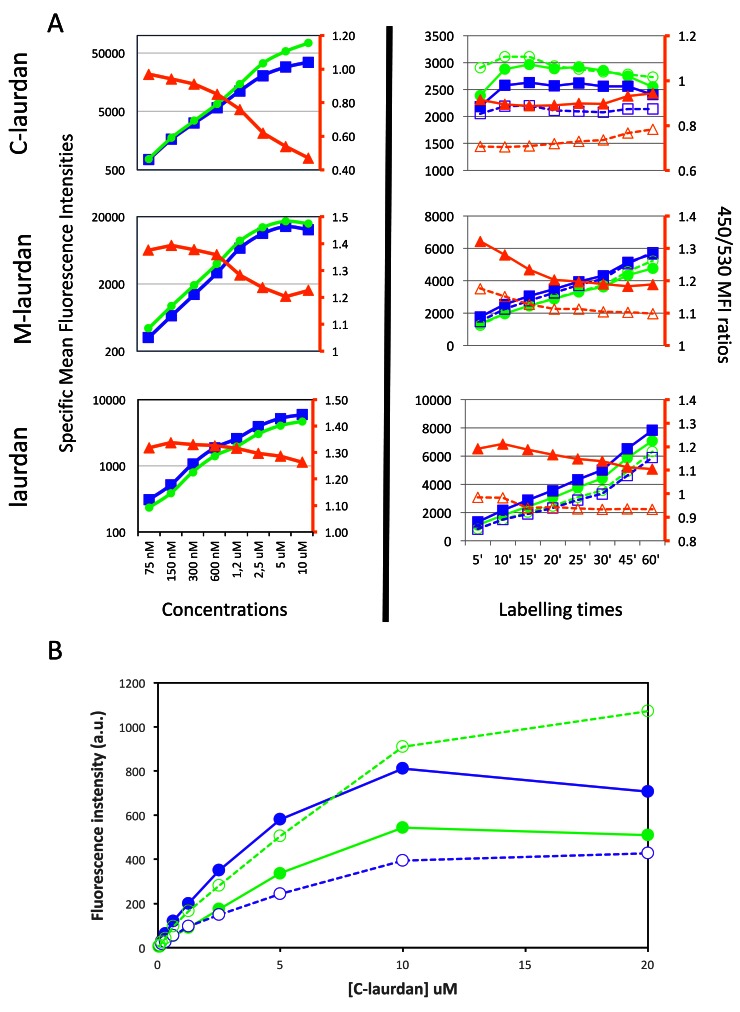
Using flow cytometry (FACS) to quantify staining intensities at 450 and 530 nm, as well as the ratio between the two. ( **A**) FACS was used to quantify the mean fluorescence intensities (MFIs) of Hela cells labelled with C-laurdan (top), M-laurdan (middle) or laurdan (bottom). Blue squares indicate MFI at 450 nm and green circles indicate MFI at 530 nm, as plotted on the y-axis on the left side of each plot. Orange triangles indicate the ratio of 450/530 MFIs as plotted on the orange y-axis on the right side of each plot. The plots in the left column show dose–response curves after labelling cells at 37°C for 60 minutes. The plots in the right column show time courses of cells labelled at 37°C with either 200 nM C-laurdan, 500 nM M-laurdan or 4 µM laurdan. The unfilled blue squares and orange triangles indicate measurements as for the filled symbols, but on cells treated with 5 mM MßCD for 30 min at 37°C before labelling. ( **B**) The preferential autoquenching of C-laurdan in Lo environments, suggested by the drop in the 450/530 ratio in the FACS analysis of cells labelled with high concentrations of the probe, can also be seen in MLVs analysed on a standard spectrofluorimeter. Serial two-fold dilutions of C-laurdan in DMSO were added (1% vol), whilst vortexing, to aliquots of MLVs prepared either with DOPC (Ld, dotted lines) or with DPPC–chol (Lo, continuous lines). After 20 min incubation at 37°C, the emission spectra of each aliquot were recorded in quartz cuvettes in a standard spectrofluorimeter (excitation: 360 nm), and the fluorescence intensities at 440 nm (blue lines) and 490 nm (green lines) were plotted against the concentration of C-laurdan.

As can be seen in Figure 8, when we incubated cells with increasing concentrations of C-laurdan for 60 min at 37°C, fluorescence intensities increased in both channels, but the 450/530 ratio decreased progressively, and very notably, for concentrations superior to 1.2 µM. When we looked at the evolution of the 450/530 ratio over time in cells labelled with 200 nM of C-laurdan, we found that this ratio was very stable over time, and, if anything, tended to go up slightly for incubation times superior to 30 minutes ( Figure 8A, upper right panel). The progressive disappearance of staining of the PM seen by microscopy at high concentrations of C-laurdan fits with the reduction in the 450/530 ratio seen by flow cytometry with increasing concentrations of the probe. Thus taken together, our results strongly suggest that, in cells kept at 37°C, C-laurdan does not have an intrinsic affinity for an intra-cellular compartment in which it would get progressively trapped. A more likely explanation seems to be that some autoquenching of the probe occurs preferentially in the PM. This more marked tendency of C-laurdan to autoquench in a Lo environment was confirmed by performing staining of MLVs with increasing concentrations of C-laurdan and measuring fluorescence in a standard spectrofluorimeter. As can be seen in Figure 8B, the fluorescence intensities recorded plateaued above 10 µM of C-laurdan, and this was even more noticeable at 440 nm than at 490 nm, with the signal at 440 nm actually decreasing for DPPC–cholesterol MLVs stained with 20 µM of C-laurdan. 

When similar experiments were performed with cells labelled either with laurdan or M-laurdan, we found evidence for some autoquenching occurring with M-laurdan at concentrations above 600 nM, but not with laurdan ( Figure 8A, middle and bottom panels). It can be noted that the tendency to autoquench correlates with the efficiency in labelling cells. In the experiment shown, the autoquenching becomes noticeable for levels of fluorescence superior to 10.000 for both C-laurdan and M-laurdan. The reason why autoquenching is not seen for laurdan may thus be that the levels of staining with that probe never attain these values.

When it came to optimizing the times necessary for staining cells, C-laurdan turned out to be a much simpler probe to use. Indeed, for C-laurdan, levels of staining and the 450/530 ratio were found to be remarkably stable between 10 and 30 min, but this was not the case for the other two probes. First, levels of staining with laurdan and M-laurdan kept increasing over time all the way to 60 minutes. Additionally, for M-laurdan the 450/530 ratios decreased during the first 20 minutes, probably as a consequence of the probe's diffusion from the surface to the intracellular membranes.

In order to validate the use of cytometry to evaluate the polar/apolar ratio in cell membranes, we performed staining of cells that had been treated with methyl beta cyclodextrin (MßCD) to deplete them of cholesterol (dotted lines and hollow symbols in right hand side panels of Figure 8A). As expected, this resulted in very significant decreases of the 450/530 ratio for all three probes, with evolutions over time that paralleled those recorded on untreated cells.

Based on these results, we elected to use 200 nM of C-laurdan, 500 nM of M-laurdan or 2 µM of laurdan as the concentrations that give the best compromise between good levels of staining and minimal autoquenching, and to incubate the cells with the probes for at least 45 min at 37°C before observation for the latter two probes, and 20 minutes for C-laurdan. We also find that maintaining the cells in the staining medium for imaging gives the best results. Of note, if rinsing needs to be performed, it should be done with medium with no serum, lest the labelling will rapidly fade away, especially for C-laurdan. But in our experience this is really not necessary since the probes are only fluorescent when inserted in a lipid environment.

Dataset 1. Intermediary flow cytometry data for Figure 8: Using flow cytometry (FACS) to quantify staining intensities at 450 and 530 nm, as well as the ratio between the two http://dx.doi.org/10.5256/f1000research.11577.d162294
Copyright: © 2017 Mazeres S et al. 2017 Data associated with the article are available under the terms of the Creative Commons Zero "No rights reserved" data waiver (CC0 1.0 Public domain dedication).

### Optimising the presentation of the results of spectral decomposition

**Figure 9.**
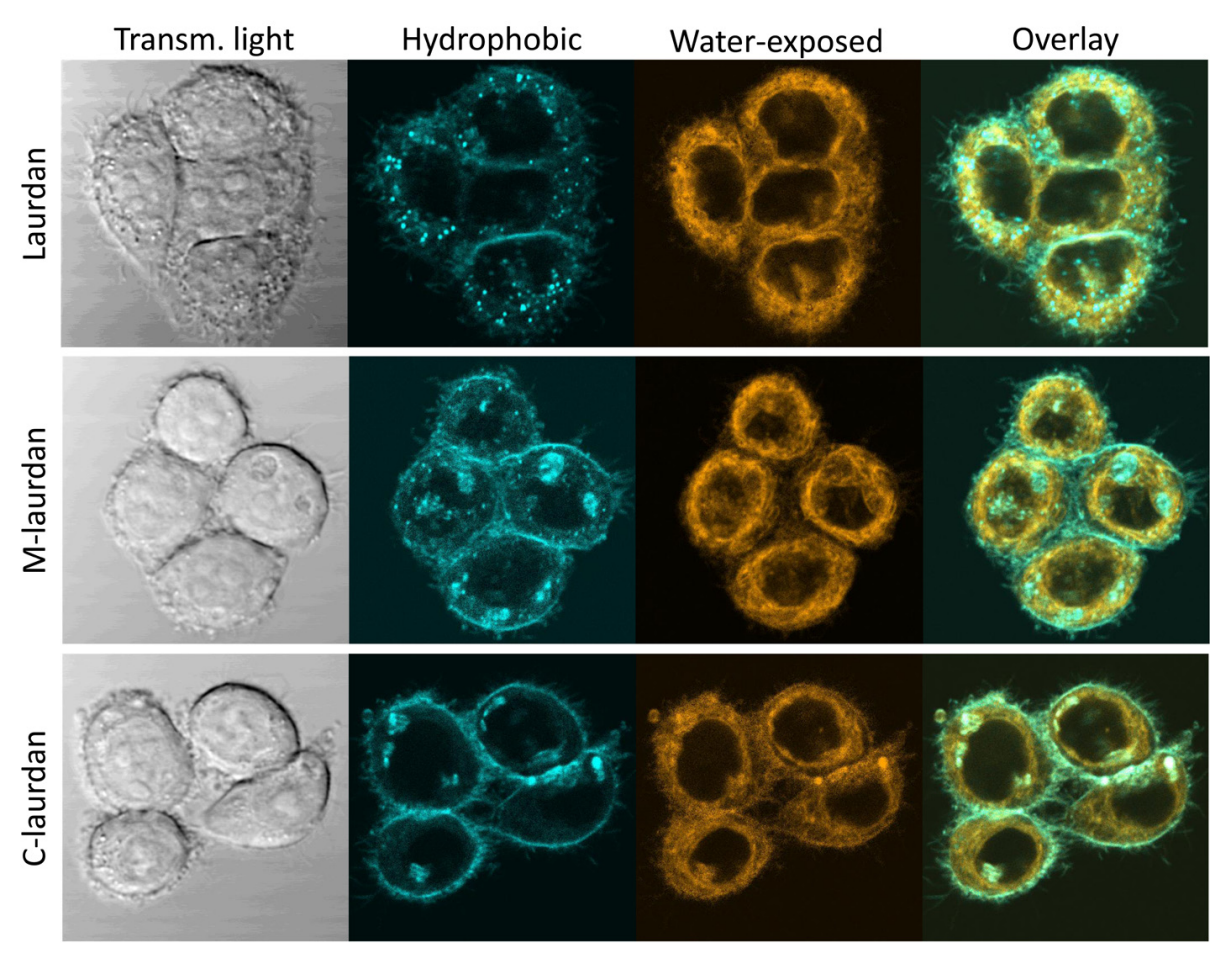
After spectral deconvolution, the staining patterns obtained with Laurdan, M laurdan and C laurdan are rather similar, but C laurdan labels the PM noticeably better than the other two probes. Live Hela cells grown on glass coverslips for two days were labelled with either 2μM Laurdan (top row), 500 nM M-laurdan (middle row) or 200 nM C-laurdan (bottom row) as described in M&M. Lambda stacks were then acquired on live cells kept at 37°C with our bi-photon microscope, and spectral deconvolution carried out, all with the standard conditions specified in M&M. Left column: transmitted light; second column (cyan): deconcolved fluorescent signal corresponding to the probes in an apolar, hydrophobic environment ; third column (orange): deconcolved fluorescent signal corresponding to the probes in a polar, water-exposed environment; fourth column: overlay of the signals in the second and third columns.

Having established the appropriate concentrations and incubation times for the three probes, we could then compare the staining patterns obtained on Hela cells after spectral decomposition. The results of the decomposition consist of two-channel stacks, which correspond, respectively, to the fraction of the probe in either apolar or more water-exposed environments. After numerous tests and surveys among users to find out what two-colour combination worked best for most people, we selected to use the cyan colour for the first channel, and orange for the second one. As can be seen on Figure 9, all three probes did result in very similar overall patterns, but with some noticeable differences: whilst laurdan tends to accumulate inside cells, and very noticeably inside lipid droplets, C-laurdan labels the PM more efficiently, and M-laurdan has an intermediate behaviour.

**Figure 10.**
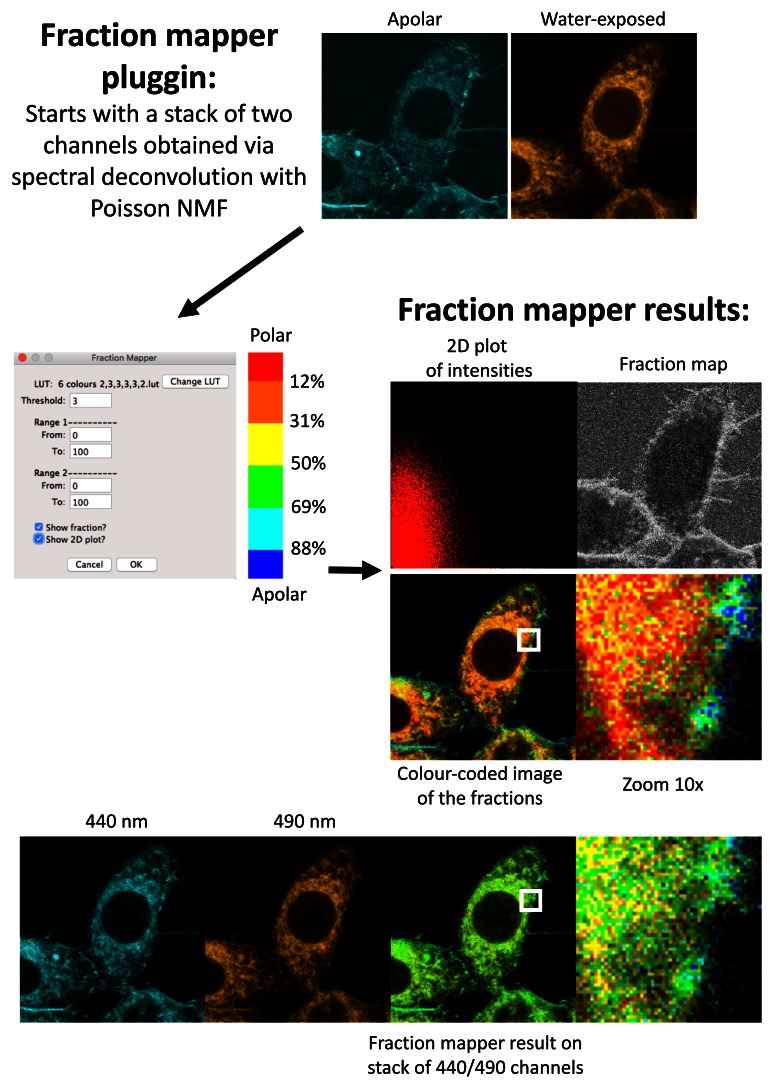
The Fraction Mapper plugin. Starting from a stack of two channels, Fraction Mapper calculates the fraction of the intensity in the first channel for every pixel (ch1/ch1+ch2), which can be obtained as a fraction map. A 2D plot of the intensities in each of the channels can also be generated. The plugin then colour-codes the numbers corresponding to the fraction according to a chosen LUT, and gives the pixels the light intensity of the sum of the two channels. The LUT used was based on six discrete colours from blue to red (LUT ‘6 colours 2,3,3,3,3,2’ available for download as Supplementary File 2). Bottom row: Starting from the 440nm and 490 nm channels, classically used for calculating GP, the picture obtained is much less informative. Of note, GP values are closely related to fraction values we use in this study: the GP can be obtained simply by multiplying the fraction by two and subtracting 1. Owen *et al*. have produced and published a ‘GP Calculator’ plugin ( Owen *et al.*, 2011) that provides very similar results to those obtained with Fraction Mapper, albeit by a more indirect approach. The 10× magnifications correspond to the 52×52 pixels regions indicated by white squares in the adjacent image; each pixel is 100×100 nm and the whole pictures cover 52×52 microns.

Whilst the cyan-orange two-colour representation is already quite informative, we found that it was not at all appropriate to evaluate the ratio of the two channels in the various cellular compartments. To this end, we generated the Fraction Mapper plugin for ImageJ, which proceeds in successive steps ( Figure 10). Starting from a two-channel image stack, such as the ones obtained with the PoissonNMF plugin, Fraction Mapper first calculates the fraction of signal in the first channel for every pixel (i.e. value of pixel in first channel / sum of values for that pixel in first and second channel). Fraction Mapper then colour-codes this fraction according to a chosen LUT, and gives each pixel the intensity of the sum of the two components. For our purpose, we find that a LUT based on six discrete colours from blue to red gives the clearest results ( Supplementary File 2). Compared to relying just on the fluorescence recorded in the 440 and 490 nm channels, as classically used when calculating GP and shown on the panels at the bottom of Figure 10, the spectral decomposition approach based on combining PoissonNMF and Fraction Mapper can clearly result in a very marked improvement of the resolution between different cellular compartments.

**Figure 11.**
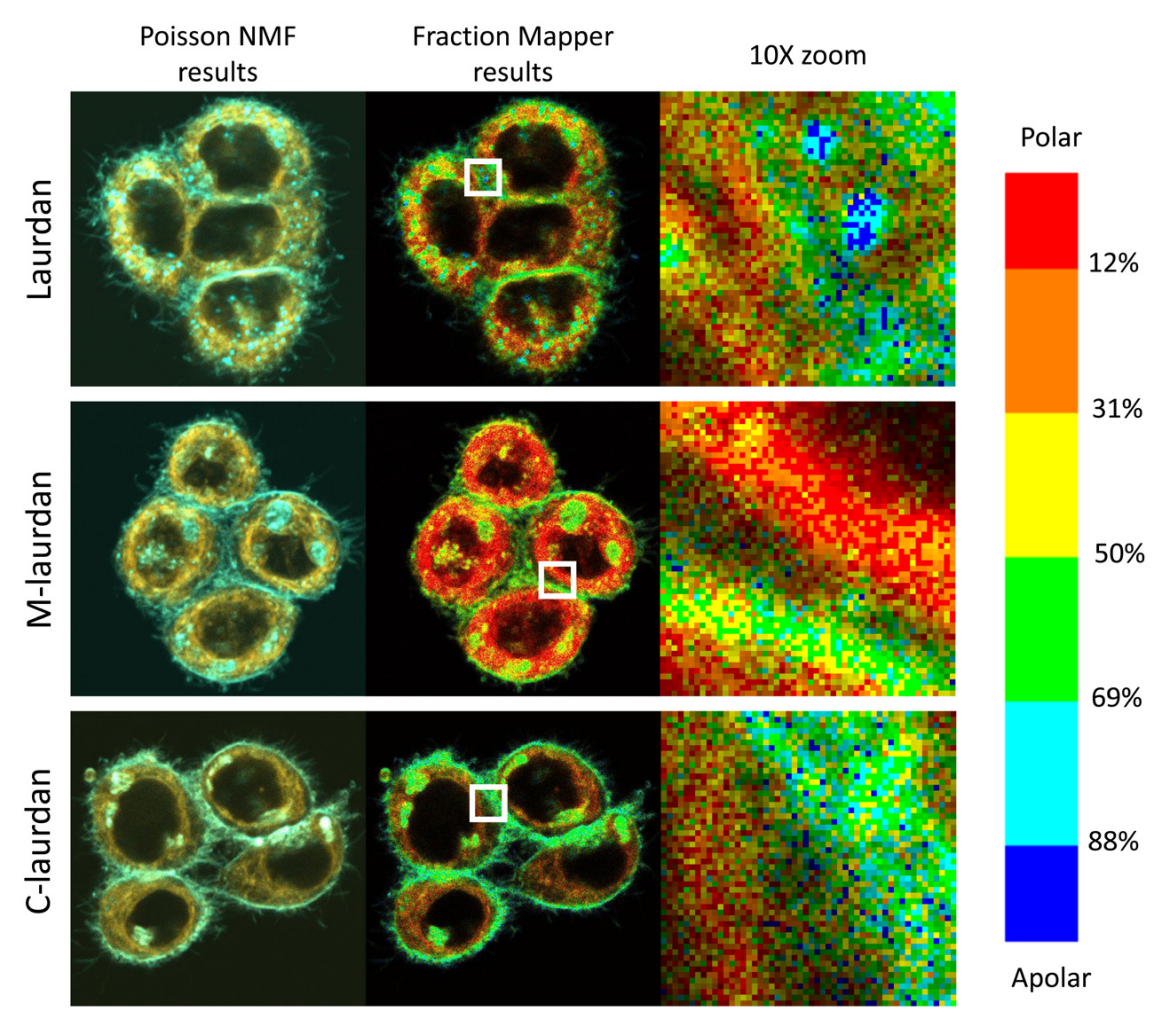
Fraction Mapper gives a much clearer picture of the relative polarity of the lipid environment in various cellular compartments than two-colour overlay. In the middle column, the Fraction Mapper plugin was used on the two-colour stacks from Figure 9 (shown in left column), which were obtained by spectral decomposition using PoissonNMF. The right column shows 10x magnifications of the 52×52 pixel regions indicated by white squares in the middle column. Each pixel is 100×100 nm and the whole pictures cover 52×52 microns. Top row, 2uM laurdan; middle row, 500 nM M-laurdan; bottom row, 200 nM C-laurdan.

With the representation obtained using Fraction Mapper, one can rapidly see that, in Hela cells stained with the three laurdan-derived probes ( Figure 11), although the distribution of the three probes is quite different, the overall picture is basically the same: intra-cellular compartments harbour a majority of water-exposed probes (yellow, orange and red), corresponding most likely to membranes predominantly in Ld states, whilst the regions near the PM show a strong dominance of apolar environments, which presumably correspond mostly to Lo states.

Upon ticking the corresponding box, Fraction Mapper will produce a ‘Fraction Map’ image, which can then be used to quantify the actual numerical values of the fraction of the first channel in a defined area of the pictures, such as individual pixels or ROIs drawn on particular regions of the cells.

Fraction Mapper can also generate 2D plots based on the intensity of each pixel in the two channels of the stacks of images used as starting material. On those 2D plots, it is possible to draw regions of interest or gates and to then use the ‘Gate to Image’ plugin tool to generate an image based on the fraction map, but containing only the pixels falling within the gate. This function was implemented because we felt that, considering the very sharp regional differences seen between the PM and the intra-cellular membranes, we might be able to identify separate populations of pixels on this type of graph, much like what can be done for cells in flow cytometry. This has, however, proven not to be the case: as long as the cells are alive and healthy, and as long as there are no over-exposed areas in the field, we observe no obvious clouds of dots, but always a regular distribution of the pixels instead. This has been true on all the numerous dot plots we examined in the course of this study, acquired on a variety of cell lines.

### Optimizing the settings for biphoton acquisition

The Fraction Mapper plugin provided us with the capacity to analyse rapidly the physical state of the cellular membranes we were imaging with much improved clarity and precision compared to the two-colour cyan-orange pictures obtained through spectral decomposition. In turn, this allowed us to adjust the settings we were using to ensure the reproducibility of our results; in other words, to make sure that we were not damaging the cells during the acquisition process. Indeed, one of the major pitfalls of using biphoton excitation lies with the very high intensities of light necessary for an efficient absorption of two photons simultaneously. This is of particular concern in live cells because this type of illumination can very easily induce cell damage, in particular at the level of membranes. For our study, which focuses on the physical state of biological membranes, the localized delivery of heat was also of particular concern to us because, as anybody who has ever left butter in direct sunshine, or tried to spread butter on toast when it comes straight out of the fridge knows only too well, the physical state of lipids is especially sensitive to changes in temperatures.

**Figure 12.**
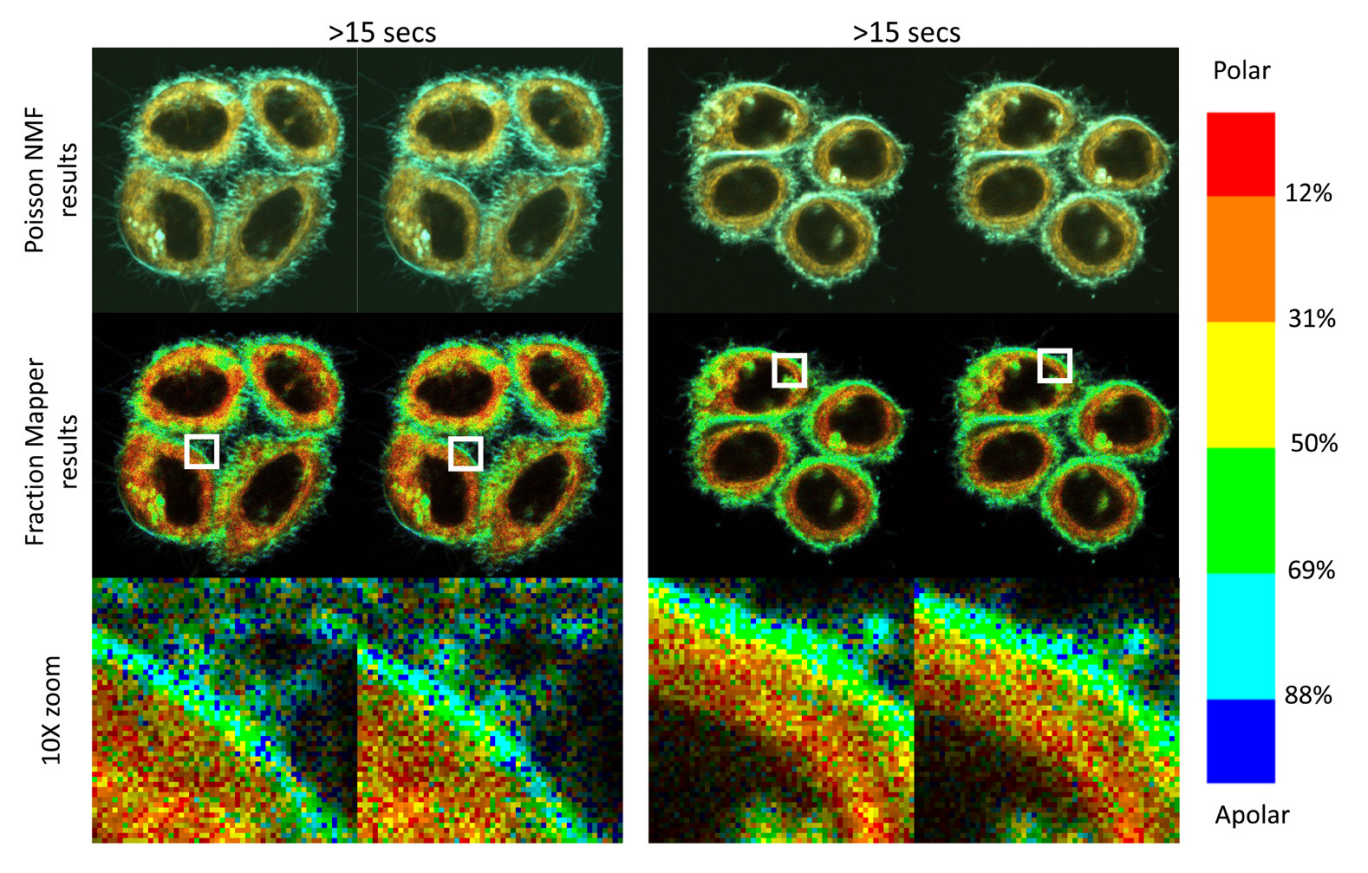
Using 2 sequential acquisitions 15 seconds apart to ensure that the acquisition has not modified the membranes or damaged the cells. Hela cells grown for two days on glass coverslips were stained with 200 nM C-laurdan and imaged twice sequentially with the standard conditions defined in M&M (power 4%, speed 6, 4 averages, pinhole 150). With these setting, the acquisition procedure takes 15 seconds. The second picture is thus taken 15 seconds after the first one. Z4:Whole image : 53 µm, 1 pixel = 100 nm HELA C Laurdan 200 nM, Laser at 720 nm, Z4, power: 4%, speed: 6 (6 usec/pixel), average on 4 measures, pinhole: 150 Hela CL 4 and Hela CL 5 15 Oct 2015.

Ultimately, we found that the systematic sequential acquisition of two pictures, coupled with the Fraction Mapper representation, was a very effective way to adjust our settings to obtain good signals, whilst ensuring that we were not inducing damage, or even just modifications to the membranes of the cells we were studying ( Figure 12). For example, we find that using laser powers over 6% can very rapidly induce membrane blebs, and we therefore never use powers over 4%. Although biphoton excitation already results in excitation that is limited to a depth of about 1 μm, this resolution along the z axis can be somewhat improved a little bit further by closing the pinhole normally used for confocal acquisition with one-photon excitation. Whilst we did find that closing the pinhole could marginally improve the resolution in the pictures, we also found that closing it past a value of 150 resulted in an excessive loss of photons, which required increasing the laser power above 4%. Other factors that we identified as possibly resulting in altered pictures between the first and the second passage are excessively long dwell times per pixel or excessive number of averages, if only because both can also result in very long acquisition times. In the end, the standard settings we have elected to use are as follows: laser power: 2–4% (corresponding to 3–6 mW average power delivered, as measured at the level of the sample), pixel dwell time: 6.3µsec, average on 4 measures, pinhole: 150. With such settings, the acquisition of a 512x512 picture takes 15 seconds, and as can be seen on Figure 12, the state of the cells remains unaltered during the second acquisition compared to the first passage, whilst the micro heterogeneities seen at the level of individual 100 nm pixels are themselves remarkably fluctuant.

### Comparing the results of spectral decomposition in a variety of cell lines

**Figure 13.**
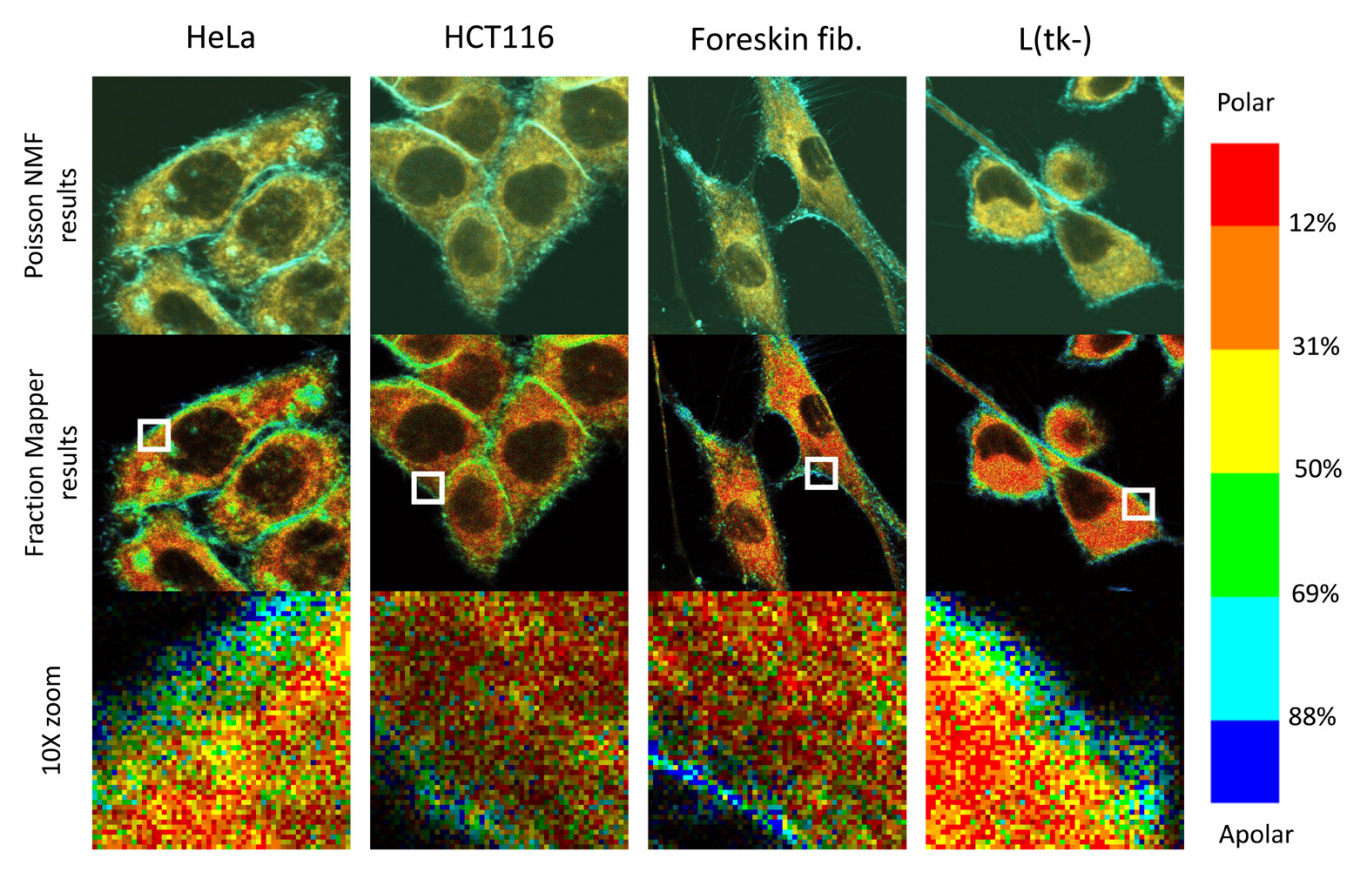
C-laurdan stains various cell types in similar patterns. Human HeLa cells (first column), HCT116 cells (second column), foreskin fibroblasts in primary culture (third column) and mouse L(tk-) cells (fourth column) were all grown on glass coverslips before staining with C-laurdan, imaging and analysing the images by the standard procedure, as described in Methods. Top row: overlay of the two channels obtained after spectral decomposition with PoissonNMF (cyan, apolar environment; orange, polar, water-exposed environment). Middle row: the two channel stacks were processed with the Fraction Mapper plugin using the six-colour 2,3,3,3,3,2 LUT shown on the right-hand side. Bottom row: 10x magnifications of the 52×52 pixel regions indicated by white squares in the middle row. Each pixel is 100×100 nm, and each picture, taken at zoom 4, covers 52×52 microns.

Having established these optimized settings and conditions using Hela cells, we turned our attention to a panel of other cell lines, and found very similar patterns in all the mammalian cells we looked at (see Figure 13 for three examples).

**Figure 14.**
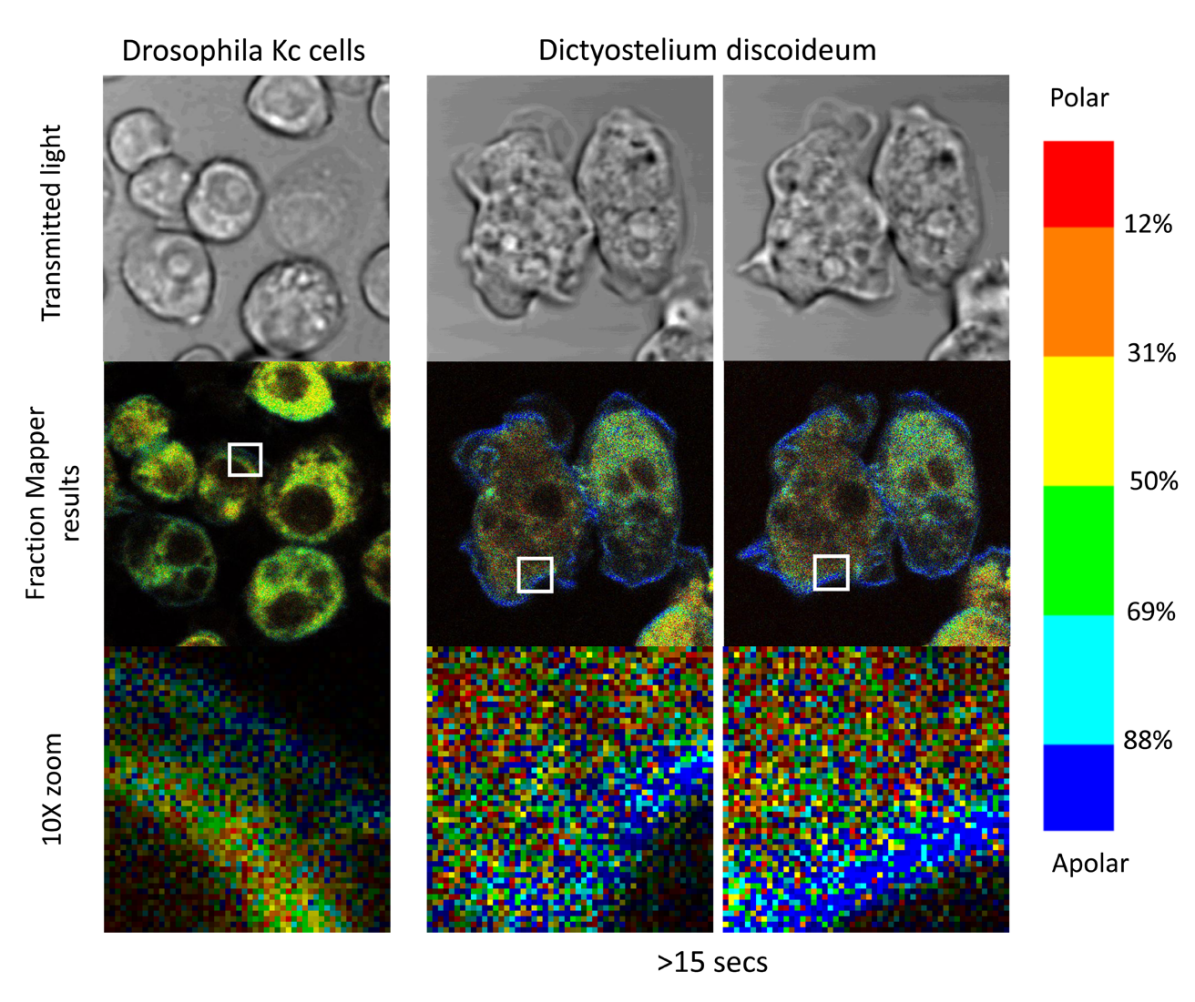
Staining of *Drosophila* and *Dictyostelium* cells. Right column: KC cells from Drosophila melanogaster were grown for 48 hours on PLL-coated coverslips in a 6-well plate before transfer to an observation chamber and staining with 400 nM of C-laurdan in serum-free medium. Middle and right columns: Dictyostelium discoideum amoebae (AX2 strain) were transferred directly to an observation chamber with a glass coverslip bottom for a couple of hours in axenic medium before staining with 800 nM C-laurdan in Sörensen’s buffer. The pictures in the right column, taken 15 seconds after those in the middle column, show that the motility of the amoebae triggered by the stimulation by light of these photo-reactive cells is not accompanied by any noticeable modification in the overall state of their lipid membranes. All pictures were taken at zoom 6: whole picture 35×35 µM, pixel size : 69 nm Droso: Tests 20 Oct 2016 Droso KC (Z6 3) Dictyo :Tests 25 Oct 2016 : AX2 Z6.

Considering the importance of temperature in regulating the state and order in lipid bilayers ( Burns *et al.*, 2017), we wondered what the situation would be in cells from ectotherms. To this end, we turned our attention to cells from *Drosophila melanogaster*, and to *Dictyostelium discoideum*, which both grow at room temperature. For both of these cell types, we found that we needed to use slightly higher levels of the C-laurdan probe to reach satisfactory levels of staining (but which did not lead to any detectable autoquenching of the probe). The fact that these organisms are less efficiently labelled by C-laurdan may be due to their lipid composition being different from that of mammalian cells, such as different sterols and lipid chains better adapted to life at more variable temperatures. In line with this hypothesis, the results shown in Figure 14 reveal that the C-laurdan probe, when inserted in the membrane compartments of these two very different organisms, tends to emit fluorescent light that is blue-shifted compared to when it is in mammalian cells: the cytoplasm appears dominantly yellow rather than red-orange, and the PMs show a high prominence of dark blue rather than cyan pixels. On the whole, C-laurdan thus seems to be slightly less exposed to water when it is inserted in the membranes of those cells than in the membrane of mammalian cells, which would suggest that lipid membranes of these ectotherm organisms tend to be a bit less permissive for water to penetrate deep into the lipid bilayers. At the cellular scale, however, the overall picture remains the same, with the environment of the PMs of the cells of these two ectotherms being much more apolar than their intra-cellular compartments.

## Discussion

The capacity to perform spectral decomposition on lambda-stack images of cells stained with solvatochromic membrane probes of the laurdan family provides much clearer results than simply relying on GP calculations based just on the 440 and 490 nm channels. The idea of making use of the additional information contained in a lambda stack acquired via a spectral analyzer compared to simply using the 440 and 490 nm channels has already been recently explored by Sezgin *et al*. (2015). Whilst the spectral imaging approach described by these authors did result in improved accuracy for the detection of differences in polarity in model and cellular membranes, they did, however, find that this approach was not very well suited for staining of live cells with C-laurdan. After attempting to use their “GP plugin”, we concur with their conclusions, and find that our approach based on spectral decomposition with PoissonNMF provides much clearer pictures, as well as being much less demanding in terms of calculating computer time.

One important point to bear in mind, however, is that, although the results we report here rely on precise computer-based mathematical calculations, the numbers obtained should only be taken as a rough indication of the actual ratio of the probes in apolar and water-exposed environments, and this, in turn, should only be considered as a rough proxy for the ratio of membranes in disordered *versus* ordered states. Among the many reasons why this type of approach cannot be rigorously quantitative, the main one is that the results of the decomposition procedure are very sensitive to the settings used, including the power of the laser, the speed and number of acquisitions, or the actual reference curves used for the decomposition process. For example, although the emission curves of the three laurdan-derived probes are very similar to one another, we found that we could get very noticeably different results if we inadvertently used the reference curves for one probe to analyse the results obtained with another probe. This seems particularly relevant given that the reference curves we used for each probe were acquired on simple DOPC and DPPC-chol MLVs (in other words on completely artificial membrane systems), and that sphingolipids, which are very prominent in biological membranes, can somewhat alter the emission spectra of those laurdan-based probes ( Bagatolli *et al.*, 1998; Mazeres *et al.*, 2014). All in all, however, based on numerous experiments performed on a whole variety of cell lines, and in line with the results of several other previous studies ( Dodes Traian *et al.*, 2012; Golfetto *et al.*, 2013; Kucherak *et al.*, 2010; Niko *et al.*, 2016; Owen & Gaus, 2010; Owen *et al.*, 2011; Yu *et al.*, 1996), we feel that we can very confidently state that, in plasma membranes, there is a very significant dominance of organized domains, whilst lipid bilayers in intra-cellular compartments are mostly in a liquid disordered state.

The degree of order inside lipid bilayers is directly linked to their local viscosity, and this can significantly influence the fluorescence lifetime of a variety of lipid fluorescent probes. Because fluorescence lifetimes are effectively independent of the probes’ concentration, imaging based on fluorescence-lifetimes, is often considered as more reliable than relying on spectral differences to perform quantitative analyses. Such approaches have used a variety of solvatochromic probes, such as laurdan ( Golfetto *et al.*, 2013; Owen & Gaus, 2010; Owen *et al.*, 2012b), di-4-ANEPPDHQ ( Owen & Gaus, 2010), F2N12S ( Kilin *et al.*, 2015), PA ( Niko *et al.*, 2016) or different molecular rotors ( Dent *et al.*, 2016) and references therein. The results of these various studies all concur to show that the PMs of eukaryotic cells are a much more organized environment (and more impermeable to water) than the membranes of the intra-cellular compartments. This is in very good agreement with the ordering properties of cholesterol on liquid organized lipid bilayers, since there is a gradient of cholesterol (or other sterols in plants, fungi or invertebrates) going from nucleus to the plasma membrane ( van Meer *et al.*, 2008). In the nuclear envelope and endoplasmic reticulum, sterols represent less than 10% of the lipids. They get progressively enriched through the Golgi apparatus, and account for more than 30% of lipids at the PM, and can even make up almost 50% of PMs’ lipids in some cases. In the reverse direction, i.e. during endocytosis, sterols get progressively eliminated from endosomes, and are virtually absent from lysosomes ( Darwich *et al.*, 2014).

Using quantitative analyses based on time-resolved lifetime imaging coupled to phasor analyses, Owen and colleagues did famously manage to evaluate that the PM of live mammalian cells was comprised of approximately three quarters of ordered lipid domains ( Owen *et al.*, 2012b). Our results appear to be in very good accordance with this previous study, and with all the others cited above. A major drawback of approaches based on fluorescence lifetime is, however, the long acquisition time inherent to the technique of time-correlated single-photon counting (TCSPC). Although our approach based on spectral decomposition and fraction map analysis may not be as rigorously quantitative as those based on time-resolved approaches, one of its major advantages lies with the speed of data acquisition. Using the standard settings described here, a whole 512×512 picture takes 15 seconds to acquire, but this could be considerably accelerated by simply reducing the size of the imaging field, and even further by shortening the pixel-dwelling time by a factor of two or three without too much effect on the noise to signal ratio. This type of approach would thus seem very well fitted for more dynamic studies, such as documenting the cellular responses to temperature shifts or to focalized stimuli.

Regarding the dominance of a disordered state of the intra-cellular membrane compartment, this is not entirely surprising since most of those membranes would be expected to correspond to the ER, where it is known that there are very low levels of cholesterol. We were, however, somewhat surprised not to observe readily detectable peri-nuclear areas of decreased polarity corresponding to the Golgi or to endosomes ( Niko *et al.*, 2016). In future work, we plan to address such questions by making use of red-fluorescent proteins targeted to various cellular compartments. With such markers, given the capacity of our system to separate red fluorescent signals from those emitted by the laurdan-derived probes (see Figure 2, Figure 3 and Figure 6), we should be in a position to explore the degree of order of various intra-cellular compartments, with a particular emphasis on the ER > Golgi > PM exocytosis axis.

In most of our pictures, the thickness of PMs appear to span over several pixels, corresponding to several hundreds of nanometers. This is far wider than the thickness of a lipid bilayer, i.e. 4–10 nm, but this is not surprising given that we are not using super-resolution microscopy. Therefore, the limit of resolution in our pictures corresponds to 0.4 times the wavelength divided by the numeric aperture of the microscope objective, i.e. 0.4×500/1.2 = 167 nm at best, corresponding to two pixels in most of our pictures. In this regard, although it may be tempting to ponder if the pixel-wide heterogeneities seen both at the level of the cytoplasm and the PM in all of our Fraction Mapper results could correspond to the famously elusive microdomains, this can simply not be the case, and those heterogeneities must thus simply result from noise, since we are at the limits of sensitivity of this type of acquisition procedure.

Another factor that can contribute to increasing the width of the membranes is that, even if the depth of the biphoton excitation is very limited (in other words the size of the voxel along the z axis), this depth is still of the order of one micron (as mentioned earlier, closing the pinhole of the confocal microscope can result in a reduction of this thickness, but with the immediate drawback of dramatically reducing the intensity of the captured light). Over such a thickness of several hundreds of nanometers, the PM is therefore very unlikely to be completely flat and perfectly aligned with the microscope’s objective. It is therefore not so surprising that the areas of high hydrophobicity that surround the cells and are expected to correspond to the PM should span multiple pixels, corresponding to several hundreds of nanometers. In addition, the intra-cellular membrane compartments that are adjacent to the PM might become superimposed with the PM, and make it look wider that it actually is. Such compartments would be expected to correspond mostly to vesicles of exocytosis coming from the Golgi, and of endocytosis coming straight from the PM ( Darwich *et al.*, 2014). Both those types of vesicles would contain significant levels of cholesterol, and hence be likely to represent more apolar environments compared to the membranes of the ER, which contain very low levels of cholesterol. On the other hand, the superimposition of mostly-disordered intra-cellular membranes with the plasma membrane would result in a reduction of its apparent order. In line with this hypothesis, in most of the pictures we have obtained, the dark blue pixels, which are expected to correspond to very ordered membranes, are mostly found on the outer edge of cells (zooms in Figure 12– Figure 14), i.e. in areas where the PM cannot be superimposed with intra-cellular membranes. Of note, outside of the presence of the high fraction of sterols, at least two other factors contribute significantly to increasing the order at the PM. First, the overall populations of lipids differ greatly between the plasma membrane and the intra-cellular compartments, with the PM containing high proportions of sphingolipids as well as phospholipids with more saturated acyl chains due to enzymatic lipid remodeling ( Bigay & Antonny, 2012; Hishikawa *et al.*, 2014; van Meer *et al.*, 2008). Second, the attachment of actin cytoskeleton anchors can also contribute to the formation of ordered domains in the PM ( Dinic *et al.*, 2013).

In turn, one may wonder how functional microdomains (aka rafts) may actually form in an environment that is already so dominantly in a liquid organized form? Whilst some have expressed the views that this may rely on the coalescence of small discontinuous domains ( Owen *et al.*, 2012b), or even to the formation of ‘reverse rafts’ ( Wassall & Stillwell, 2009), another possibility lies with the formation of even more densely organized domains ( de Almeida & Joly, 2014; Joly, 2004).

## Software and data availability

Source code for the Fraction Mapper plugin: https://github.com/farzadf58/FractionMapper/tree/1.0

Archived source code as at time of publication: doi, 10.5281/zenodo.581815 ( Fereidouni, 2017)

License: MIT

Dataset 1: Intermediary flow cytometry data for Figure 8: Using flow cytometry (FACS) to quantify staining intensities at 450 and 530 nm, as well as the ratio between the two. doi, 10.5256/f1000research.11577.d162294 ( Mazeres *et al.,* 2017).

## Supplementary material

**Supplementary File 1: Film S1:** In live Hela cells kept at 37°C, the intra-cellular vesicular bodies labelled by laurdan are very mobile. Hela cells were grown on coverslips for two days before labelling them with laurdan and imaging in lambda mode with our biphoton microscope as described in Methods. 10 successive pictures were taken every 30 seconds. The channels centred on 442 nm (left panel), 491 nm (center panel) and that for transmitted light were then extracted from the resulting stack, and recombined to create this movie. The fact that the vesicles are much more visible at 442 nm than at 491 nm suggests that they correspond to a very hydrophobic environment.

**Supplementary File 2: 6 colours 2,3,3,3,3,2.lut.** Look up table for the presentation of Fraction Mapper results. This LUT is based on six discrete colours from blue to red.

**Supplementary File 3: Rainbeau.lut.** Look up table used for recoloring the image stacks acquired in lambda mode over 29 channels between 418 nm and 700 nm.

Practical examples with step by step Instructions (PDF document + zipped folder with all the necessary files, plugins and luts)
