# Supplementary material for: Using spectral decomposition of the signals from laurdan-derived probes to evaluate the physical state of membranes in live cells
Source: F1000Res. 2017 Aug 1;6:763. Originally published 2017 Jun 1. [Version 2] doi: 10.12688/f1000research.11577.2 (PMC5473435; doi:10.12688/f1000research.11577.2)
Supplement: Supplementary file 5 [file f1000research-6-13200-s0004.tgz › 55373b50-d94d-4a93-b797-350603cc4694.zip!/Step_by_step_instructions_for_spectral_unmixing.pdf]

**Plugins installation**

Before starting, make sure to install the following plugins and files

- **The Poisson NMF plugin** can be downloaded from https://github.com/neherlab/PoissonNMF as a 15 Mb zip file which contains a variety of programs and example files. From this, all you will need is the PoissonNMF_.jar file found in Documentation>NMF folder. You can also find this file included in the “Step by step tutorial” folder provided.
  To install the plugin, copy the **PoissonNMF_.jar** file into the plugin folder of your ImageJ distribution, together with the **SpectraLibrary** folder provided with these instructions, which already contains the four spectra that can be extracted from the files provided. Once done, restart ImageJ. The PoissonNMF line should appear in the plugin pull-down menu.

- **The Fraction Mapper plugin** can be downloaded from https://github.com/farzadf58/FractionMapper
  Or simply use the copy placed in the “Step by step tutorial” folder provided. Open the Fraction mapper 1.1 folder and copy the tree .class files as well as the Blablab folder into the plugins folder of ImageJ. Once done, restart ImageJ. The PoissonNMF line should appear in the plugin pull-down menu.

- Place a copy of the files “Zen Orange.lut” and “6 colours 2,3,3,3,3,2.lut” provided with these instructions in the luts folder of the ImageJ application.

- If you decide to use the **Action Bar plugin**, you can download it from the download section of the page at the end of the following link http://imagejdocu.tudor.lu/doku.php?id=plugin:utilities:action_bar:start
  Or simply copy the folder called ActionBar placed in the “Step by step tutorial” folder provided in your ImageJ plugins folder. Once done, restart ImageJ. The ActionBar line should appear in the plugin pull-down menu.

**Step by step instructions for carrying out spectral decomposition from 2 separate probes**
(Laurdan and Nile Red in the example provided)

A. Extracting the emissions spectra from files obtained from cells stained with only one probe.
   1) Open the file "HELA laurdan lambda Trans 02.lsm" with ImageJ
   2) Remove slice number 30, which is the one for transmitted light
   3) Chose PoissonNMF from the plugin menu
   4) Select 1 source, type OK
   5) Select the following settings: iterations: 10, subsamples: 2, segregation bias: 0, saturation threshold: 4000, background threshold: 50, Background spectrum: Minimal values, Dye 1 initial spectrum: Gaussian, boxes for keeping spectrum fixed and specifying spectral channels unticked (see note at the end of this document about the reasons for not bothering to specify the spectral channels).
   6) Click "Save spectra" in the PoissonNMF Results box. Chose a name such as "Laurdan Hela 29 channels.emn". The spectrum will be saved in the SpectraLIbrary folder within the plugins folder of the ImageJ program.
   7) Close all the opened ImageJ files without saving
   8) Repeat above steps 1-7 starting with "HELA Nile Red Lambda Trans 01.lsm", calling the emission spectrum file something like "Nile Red Hela 29 channels.emn".

   Note: The same principle applies for extracting spectra from liposomes. For those, however, we collected emission spectra from 6 to 10 separate liposomes, transferred the data to Excel and pasted the average back into an .emn file to use as the reference emission spectrum.

B. Performing unmixing based on two emission spectra
   1) Open the file "HELA laurdan Nile Red lambda trans 02.lsm" with ImageJ
   2) Remove slice number 30, which is the one for transmitted light
   3) Run the plugin PoissonNMF, with the following options:
      number of sources: 2; iterations: 10; subsamples: 2; Segregation bias: 0; Saturation Threshold: 4000; Background Threshold: 50, Background spectrum: Minimal values.
      Dye 1: select "Laurdan Hela 29 channels.emn" from the pull-down menu
      Dye 2: select "Nile Red Hela 29 channels.emn" from the pull-down menu
      Tick the boxes to keep the spectra for both dyes fixed.
      Leave "Specify Spectral Channels" unticked, then click "Run!"
   4) The Poisson plugin should generate three windows:
      - NMF sources, which is the two-slice stack corresponding to the signals allocated to each of the two probes.
      - Poisson NMF spectra, showing the two spectra used for the unmixing (See note at the end of this document to see why the wavelength axis of the graph incorrectly shows 480 and 650 nm as upper and lower limits of the spectra).
      - Poisson NMF Results, from which one can obtain various informations, including a green/red overlay picture of the two channels, and a Background map
   5) The NMF sources window can then be used to generate a colored overlay such as the ones shown on figure 2 and 3 of the paper.

**Step by step instructions for carrying out unmixing of the two spectral components of C laurdan**

A. Unmixing the apolar and water-exposed components of C laurdan.
   1) Open the file "HELA C laurdan 5.lsm" with ImageJ
   2) Make a substack of the slices 1-17 of frame 1
   3) Run the PoissonNMF plugin with the following settings:
      number of sources: 2; iterations: 10; subsamples: 2; Segregation bias: 0; Saturation Threshold: 4000; Background Threshold: 50, Background spectrum: Minimal values.
      Dye 1: select "C laurdan DPPCchol 17 channels.emn" from the pull-down menu
      Dye 2: select "C laurdan DOPC 17 channels.emn" from the pull-down menu
      Tick the boxes to keep the spectra for both dyes fixed.
      Leave "Specify Spectral Channels" unticked, then click "Run!"
   4) The Poisson plugin should generate three windows:
      - NMF sources, which is the two-slice stack corresponding to the signals allocated to each of the two components of C laurdan fluorescence.
      - Poisson NMF spectra, showing the two spectra used for the unmixing (See note at the end of this document to see why the wavelength axis of the graph incorrectly shows 480 and 650 nm as upper and lower limits of the spectra).
      - Poisson NMF Results, from which one can obtain a green/red overlay picture of the two channels, and a Background map
   5) The NMF sources window can then be used to generate a colored overlay such as the ones shown on the figures 7, 9, 11, 12, 13 of the paper. The orange color we use comes from the "Zen Orange.lut" provided with the other files in the "Step by step tutorial" folder.

B. Using Fraction Mapper to represent the results of the spectral decomposition obtained by poissonNMF .
   1) Start from an 'NMF source' stack obtained as above, or from any two-image stack that can be opened in ImageJ.
   2) Select Fraction Mapper from the plugins menu
   3) Select the following settings:
      Threshold: 3; Range 1: 0 to 100; Range 2: 0 to 100 (not critical); Tick boxes for Show fraction and Show 2D plot.
      Select LUT: "6 colours 2,3,3,3,3,2.lut"
   4) Press OK
   5) The Fraction Mapper plugin should generate three windows:
      - "F- name of starting stack". This is the fraction image, where the intensity of each pixel is proportional to the fractional intensity of the first channel over the sum of the two channels.
      - "Fraction Map-name of starting stack date and time". In this Fraction Mapper image, the colors of the pixels correspond to the fractions in the F image, according to the lut chosen (here the "6 colours 2,3,3,3,3,2.lut"), and their intensity corresponds to the sum of the values in the two channels. This is the type of picture which is shown in the figures 10, 11, 12, 13 and 14 of our paper. The brightness of the pictures can be enhanced by simply clicking Auto in the adjust brightness/contrast menu.
      - "2D plot- name of starting stack", which is a 2D plot of the two channels, with each pixel represented by a single dot. If you draw a gate in this plot, you can then use the gate_to_image plugin to show the pixels corresponding to these dots on the original image.

**Using Action Bar to accelerate the analyses.**

When analyzing large numbers of files, we find that using the Action Bar plugin can accelerate the process considerably. In the “Unmixing_CL.ijm” example provided in the “Step by step tutorial” folder, we have placed a selection of our favorite one-click commands for the following actions.

| Button | Action |
|---|---|
| Frame extract | Generate substack of one frame with the option of removing the last slide for transmitted light |
| Poisson NMF | Run PoissonNMF to unmix the C laurdan components, using the default values specified above and the “C laurdan DPPCchol 17 channels.emn” and “C laurdan DOPC 17 channels.emn” as reference spectra. |
| Colour Cyan Orange | Turns a two-slices stack into a cyan-orange overlay with auto-adjusted contrast and copies the result to the computer’s memory |
| Fraction Mapper | Runs the Fraction Mapper plugin using the “6 colours 2,3,3,3,3,2.lut” and the default values specified above. Does not generate the fraction or 2D images. Copies the fraction map with auto-adjusted contrast to the computer’s memory |
| Square 52 pix | Generates a square that is 52 pixels wide (i.e. close to 1/10 of a 512x512 pixels image) |
| Copy to system | Copies the contents of the active window to the computer’s memory without having to use the pull-down menu |
| Close All Windows (Without Saving) | Does what is says |

If other actions, or other settings are desired, the macros triggered by the buttons can be very easily modified by using the ‘edit macro’ built-in command of ImageJ.

**Note on specifying spectral channels in Poisson NMF:** For the work described in the manuscript, when using PoissonNMF, we painstakingly specified the spectral channels to match those of our bi-photon microscope rather than using the default settings of the plugin, i.e. 480 and 650 nm as artificial upper and lower limits of the spectra. Specifying the spectral channels can become particularly cumbersome because, although the PoissonNMF plugin saves the settings for the next use, it also automatically reverts to the default values if there is any mismatch between the number of channels specified and the actual number of slices in the stack (for example if one forgets to remove the channel for transmitted light). A shortcut to re-specify the spectral channels consists in making of backup copy of the file of ImageJ preferences after the plugin has run at least once with the spectral channels correctly specified. When analyses with different number of channels are to be performed, or in case of a wrong manipulation having reset the preferences to default values, the backup copies of the ImageJ preferences can be used to replace the existing one before starting the ImageJ program.

In the process of writing these step-by-step instructions, we came to realize that it is much simpler to leave the Poisson NMF plugin use its default values. As long as the reference spectra have been extracted in the same manner, this has absolutely no influence on the results of the unmixing procedure. The only difference is in the picture of the spectra such as the one which appears in figure 6 of our paper, where the wavelength axis will show aberrant values. But it is much easier to modify this afterwards than to specify the spectral values within the plugin.
