# Supplementary material for: Using spectral decomposition of the signals from laurdan-derived probes to evaluate the physical state of membranes in live cells
Source: F1000Res. 2017 Aug 1;6:763. Originally published 2017 Jun 1. [Version 2] doi: 10.12688/f1000research.11577.2 (PMC5473435; doi:10.12688/f1000research.11577.2)
Supplement: Peer review history

# Peer review: Referee response for version 1

**DOI:** 10.5256/f1000research.12507.r23159

**Published:** 2017-06-13

**Authors:** Bruno Antonny

**Affiliations:** 1 Institute of Molecular and Cellular Pharmacology, University of Nice Sophia Antipolis, Valbonne, 06560, France

This is a very careful study of the physical states of cellular membranes of different organisms under  well defined and physiological conditions (e.g . temperature). The experimental approaches are well explained and performed in the most precise manner. This type of analysis, together with the recent study performed by Niko, Klymchenko and coauthors (Scientific Reports) using a different probe, provide a clear cut and most informative picture of the geography of cell membranes and should be very useful in the future for membrane biologists. Clearly, the plasma membrane differs from intracellular membranes by its high order state. As simple as it may seem, this result is fundamental and has been overlooked in the past as compared to other ideas and models of cell membrane organization, which are compatible, but which put the emphasis on nanodomains, which remain difficult to capture. Note that in addition to the cholesterol gradiant, other parameters could also contribute to the increase in lipid ordering along the secretory pathway; most notably lipid remodeling, where the acyl chains of some bulk lipids tend to become more saturated at the PM as compared to organelles of the early secretory pathway. See references below.

I have read this submission. I believe that I have an appropriate level of expertise to confirm that it is of an acceptable scientific standard.

## References

[1] KeenanTWMorréDJ: Phospholipid class and fatty acid composition of golgi apparatus isolated from rat liver and comparison with other cell fractions. *Biochemistry*.1970;9(1) :19-25 431239010.1021/bi00803a003

[2] SchneiterRBrüggerBSandhoffRZellnigGLeberALamplMAthenstaedtKHrastnikCEderSDaumGPaltaufFWielandFTKohlweinSD: Electrospray ionization tandem mass spectrometry (ESI-MS/MS) analysis of the lipid molecular species composition of yeast subcellular membranes reveals acyl chain-based sorting/remodeling of distinct molecular species en route to the plasma membrane. *J Cell Biol*.1999;146(4) :741-54 1045901010.1083/jcb.146.4.741PMC2156145

[3] BigayJAntonnyB: Curvature, lipid packing, and electrostatics of membrane organelles: defining cellular territories in determining specificity. *Dev Cell*.2012;23(5) : 10.1016/j.devcel.2012.10.009 886-95 10.1016/j.devcel.2012.10.009 23153485

[4] HishikawaDHashidateTShimizuTShindouH: Diversity and function of membrane glycerophospholipids generated by the remodeling pathway in mammalian cells. *J Lipid Res*.2014;55(5) : 10.1194/jlr.R046094 799-807 10.1194/jlr.R046094 24646950PMC3995458

# Response

**Published:** 2017-07-24

**Authors:** Etienne Joly

**Affiliations:** Equipe de Neuro-Immunogénétique Moléculaire (ENIGM), Bâtiment CNRS, IPBS CNRS Université Paul Sabatier, France

**Bruno Antonny wrote**

 “Note that in addition to the cholesterol gradient, other parameters could also contribute to the increase in lipid ordering along the secretory pathway; most notably lipid remodeling, where the acyl chains of some bulk lipids tend to become more saturated at the PM as compared to organelles of the early secretory pathway.”

***Authors’ reply:*** *We thank Dr Antonny for his appreciation of our work and for his useful suggestion. We have included an appropriate reference in the updated version of our manuscript*

# Peer review: Referee response for version 1

**DOI:** 10.5256/f1000research.12507.r23165

**Published:** 2017-06-05

**Authors:** Erdinc Sezgin

**Affiliations:** 1 Weatherall Institute of Molecular Medicine, University of Oxford, Oxford, UK

In this article, Mazeres *et al. *report a new way (spectral decomposition) of analysing the spectral imaging data to obtain a quantitative measure of the lipid organisation in plasma and internal membranes. The authors also provide an open-access image analysis platform compatible with ImageJ.

 The manuscript is well-written and explanatory. The focus of the manuscript is not novel aspects of the membrane organisation, it is rather the methodology which is quite useful in my opinion.

 I recommend publication after minor revision. Here are my specific comments:

 In the introduction; there are statements;

- “…laurdan derivatives have no preference for ordered and disordered domains…”
  This is not exactly accurate, the partitioning of these probes has been tested in GUVs and it is probably true in GUV systems they were tested, however, recently it has been shown that partitioning of the membrane probes is highly governed by the lipid packing of the coexisting domains (Sezgin *et al.*, 2015 ^1^), and Laurdan signal predominantly comes from ordered domains of cell derived vesicles (Sezgin *et al.,* 2014 ^2^). Therefore, I would formalise this sentence a bit differently so that it is not misleading.
- “The most widely accepted model to explain the ‘red shift’ of laurdan from an emission maximum of 440 nm to a maximum of 490 nm in more polar environments is based on the reorientation, or relaxation, of structured water molecules in the direct vicinity of the probe”
   believe the photophysics behind this spectral shift is a bit more complicated and would be nice to mention this and refer the reader to appropriate reports such as the nice review by Amaro *et al.*, 2014 ^3^
- “The ANEPPDHQ, Nile Red and pyrene-derived probes, by contrast, undergo more progressive shifts in their emission maxima depending on the dielectric constants of their environments. Furthermore, the shifts in emission spectra of these probes are not necessarily linked to the degree of order of the bilayers.”
  Here, the statements need references. The original paper of di-4-ANEPPDHQ paper (Jin *et al.*, 2006 ^4^) as well as Sezgin *et al.*, 2014 ^2^ show the progressive shift of these dyes. And a recent report by Amaro *et al.*, 2017 ^5^ shows the complicated spectral shift of ANEP dye unlike a robust response of Laurdan to molecular order.
- “C-laurdan, a probe first synthesised and characterised in 2007 was derived from laurdan by adding a carboxyl group to the polar head, and labels the PM of eukaryotic cells more effectively than laurdan.”
  Here the original C-Laurdan should be cited (Kim *et al.*, 2007) ^6^
- From the Materials and Methods, I could not get what is the excitation wavelength of NileRed?
- Authors should comment on whether Fraction Mapper can read any spectral imaging file? (lsm, czi, lif etc)
- In Figure 2, there is significant “yellow signal”. The conclusion of the colocalisation is very qualitative, I suggest authors to perform pearson correlation to have quantitative measure of the colocalisation (Fig 2 and 3).
- Would be very useful to write the excitation wavelength of the probes in the figure legends as well as emission bands.
- Also, it would be extremely useful to have the figures a bit more informative and less dependent to the legend. In the current form, I had to go back and forth between the figure and the legend many times to understand the figure. Instead, if the authors put a bit of descriptive text on the figures, it would have been a lot easier to understand the figures (like they did for Figure 7 where they 3min, 15 min and 30 min as well as 4 uM and 200 nm on the figure itself). For instance, for figure 2, I would write; Laurdan, M-Laurdan, C-Laurdan on the rows and “brightfield”, “probe”, “lysotracker”, “merge” and “close-up” for the columns. Similarly I would do it for every figure so as soon as the reader looks at the figure, she can get what the figure tells without reading the legend. Similarly, they should add labels to Figure 5 and 8 on the figure what thin and bold lines or colors etc mean.
- Authors should comment on possible FRET between Laurdan and NileRed when they do the spectral decomposition between these two.
- Authors mention the “autoqueching” because of the decrease in signal with concentration. Are the authors sure that this is what happens? Can that be something else?
- 3min pictures in Fig7 is different than the rest, why?
- For Figure 10-13, it is necessary to show some quantification, qualitative assessment with the colors isn’t enough.
- “Considering the importance of temperature in regulating the state and order in lipid bilayers….” Authors should give a reference here such as Burns *et al.*, 2017 ^7^.
- In the first paragraph of the Discussion, authors should also discuss the recently published improved approach of spectral imaging analysis (Aron *et al.*, 2017 ^8^)
- In the discussion, when authors discuss the differences of lipid packing, they only consider the lipid composition. They should also add a short discussion on the presence of actin cytoskeleton for plasma membrane (and absence of it for intracellular membranes) may be responsible for relatively more ordered plasma membrane compared to intracellular ones (Dinic *et al.*, 2013 ^9^).

I have read this submission. I believe that I have an appropriate level of expertise to confirm that it is of an acceptable scientific standard.

## References

[1] SezginEGutmannTBuhlTDirkxRGrzybekMCoskunÜSolimenaMSimonsKLeventalISchwilleP: Adaptive lipid packing and bioactivity in membrane domains. *PLoS One*.2015;10(4) : 10.1371/journal.pone.0123930 e0123930 10.1371/journal.pone.0123930 25905447PMC4408024

[2] SezginESadowskiTSimonsK: Measuring lipid packing of model and cellular membranes with environment sensitive probes. *Langmuir*.2014;30(27) : 10.1021/la501226v 8160-6 10.1021/la501226v 24905799

[3] AmaroMŠachlRJurkiewiczPCoutinhoAPrietoMHofM: Time-resolved fluorescence in lipid bilayers: selected applications and advantages over steady state. *Biophys J*.2014;107(12) : 10.1016/j.bpj.2014.10.058 2751-60 10.1016/j.bpj.2014.10.058 25517142PMC4269770

[4] JinLMillardACWuskellJPDongXWuDClarkHALoewLM: Characterization and application of a new optical probe for membrane lipid domains. *Biophys J*.2006;90(7) : 10.1529/biophysj.105.072884 2563-75 10.1529/biophysj.105.072884 16415047PMC1403187

[5] AmaroMReinaFHofMEggelingCSezginE: Laurdan and Di-4-ANEPPDHQ probe different properties of the membrane. *Journal of Physics D: Applied Physics*.2017;50(13) : 10.1088/1361-6463/aa5dbc 10.1088/1361-6463/aa5dbc PMC580204429449744

[6] KimHMChooHJJungSYKoYGParkWHJeonSJKimCHJooTChoBR: A two-photon fluorescent probe for lipid raft imaging: C-laurdan. *Chembiochem*.2007;8(5) : 10.1002/cbic.200700003 553-9 10.1002/cbic.200700003 17300111

[7] BurnsMWisserKWuJLeventalIVeatchSL: Miscibility Transition Temperature Scales with Growth Temperature in a Zebrafish Cell Line. *Biophys J*.2017; 10.1016/j.bpj.2017.04.052 10.1016/j.bpj.2017.04.052 28552311PMC5607031

[8] AronMBrowningRCarugoDSezginEBernardino de la SernaJEggelingCStrideE: Spectral imaging toolbox: segmentation, hyperstack reconstruction, and batch processing of spectral images for the determination of cell and model membrane lipid order. *BMC Bioinformatics*.2017;18(1) : 10.1186/s12859-017-1656-2 254 10.1186/s12859-017-1656-2 28494801PMC5427590

[9] DinicJAshrafzadehPParmrydI: Actin filaments attachment at the plasma membrane in live cells cause the formation of ordered lipid domains. *Biochim Biophys Acta*.2013;1828(3) : 10.1016/j.bbamem.2012.12.004 1102-11 10.1016/j.bbamem.2012.12.004 23246974

# Response

**Published:** 2017-07-24

**Authors:** Etienne Joly

**Affiliations:** Equipe de Neuro-Immunogénétique Moléculaire (ENIGM), Bâtiment CNRS, IPBS CNRS Université Paul Sabatier, France

We are very grateful to Dr. Sezgin for his careful evaluation of our manuscript and for his constructive comments and suggestions to improve it. We have followed most of them, or have explained below why we chose not to.

 In this article, Mazeres *et al. *report a new way (spectral decomposition) of analysing the spectral imaging data to obtain a **quantitative measure** of the lipid organisation in plasma and internal membranes.

***Authors’ comment***: *As discussed extensively in the manuscript, we suggest that the results of the approach we describe should only be considered as semi-quantitative.*

 Here are my specific comments:

 In the introduction; there are statements;

- “…laurdan derivatives have no preference for ordered and disordered domains…”
  This is not exactly accurate, the partitioning of these probes has been tested in GUVs and it is probably true in GUV systems they were tested, however, recently it has been shown that partitioning of the membrane probes is highly governed by the lipid packing of the coexisting domains (Sezgin *et al.*, 2015 ^1^), and Laurdan signal predominantly comes from ordered domains of cell derived vesicles (Sezgin *et al.,* 2014 ^2^). Therefore, I would formalise this sentence a bit differently so that it is not misleading.

***Authors reply*** *: Following this comment, we went back to re-read those papers to see if we had missed anything relating to the unequal partitioning of Laurdan or C-laurdan between ordered and disordered domains, but could not find any definite evidence *relating to partitioning of Laurdan or C-laurdan *in either of these papers. Incidentally, we also noticed that C-laurdan was used at 4uM in the 2015 study, which our data suggests would probably lead to very significant auto-quenching, preferentially in organized domains, and render any quantitative analysis meaningless.*

- “The most widely accepted model to explain the ‘red shift’ of laurdan from an emission maximum of 440 nm to a maximum of 490 nm in more polar environments is based on the reorientation, or relaxation, of structured water molecules in the direct vicinity of the probe”
  I believe the photophysics behind this spectral shift is a bit more complicated and would be nice to mention this and refer the reader to appropriate reports such as the nice review by Amaro *et al.*, 2014 ^3^

***Authors reply*** *: In the review by Amaro et al, the authors’ conclusion is: « Taking all the above facts together, one can conclude that Laurdan τr reflects the rearrangement of hydrated sn-1 carbonyls of a lipid bilayer in the liquid crystalline phase, whereas Δν mirrors the polarity, which is related to the hydration level of sn-1 carbonyls.”*

*To us, this is just a more elaborate way (not to say complicated) to say the same thing as what we stated in our paper, i.e. that the red shift is based on the reorientation, or relaxation, of structured water molecules in the direct vicinity of the probe.*


*   *

- “The ANEPPDHQ, Nile Red and pyrene-derived probes, by contrast, undergo more progressive shifts in their emission maxima depending on the dielectric constants of their environments. Furthermore, the shifts in emission spectra of these probes are not necessarily linked to the degree of order of the bilayers.”
  Here, the statements need references. The original paper of di-4-ANEPPDHQ paper (Jin *et al.*, 2006 ^4^) as well as Sezgin *et al.*, 2014 ^2^ show the progressive shift of these dyes. And a recent report by Amaro *et al.*, 2017 ^5^ shows the complicated spectral shift of ANEP dye unlike a robust response of Laurdan to molecular order.

***Authors reply*** *: We thank Dr. Sezgin for his suggestion and have included the references in the updated version of our manuscript.*


*  *

- “C-laurdan, a probe first synthesised and characterised in 2007 was derived from laurdan by adding a carboxyl group to the polar head, and labels the PM of eukaryotic cells more effectively than laurdan.”
  Here the original C-Laurdan should be cited (Kim *et al.*, 2007) ^6^

***Authors’ reply*** *: This reference was inadvertently deleted at some stage during the writing of the paper and will be re-inserted in the updated version of our manuscript.*


*  *

- From the Materials and Methods, I could not get what is the excitation wavelength of NileRed?

***Authors’ reply:*** *As was already stated in the paper, the bi-photon excitation was carried out at 720 nm. Whilst this is clearly not the optimal wavelength for Nile Red or LysoTracker Red DND-99, it still resulted in very efficient excitation of those red probes. For Nile Red, the concentration actually had to be diluted tenfold to give signal intensities similar to those of the laurdan-based probes. We have now added a sentence in the results section to clarify and emphasize this point.*


*  *

- Authors should comment on whether Fraction Mapper can read any spectral imaging file? (lsm, czi, lif etc)

***Authors’ reply:*** *As clearly stated in the manuscript, Fraction Mapper does not read spectral imaging files, but stacks of two images. As long as ImageJ can open such stacks, one should be able to run Fraction Mapper on them. If not, the stacks should simply be converted to standard TIFF format beforehand.*


*  *

- In Figure 2, there is significant “yellow signal”. The conclusion of the colocalisation is very qualitative, I suggest authors to perform Pearson correlation to have quantitative measure of the colocalisation (Fig 2 and 3).

***Authors’ reply:*** *Dr. Sezgin is absolutely right: the analysis is indeed purely qualitative, but we feel that the results are sufficiently clear and the conclusions clear-cut that there is really no need for complicated mathematical analyses that would bring nothing additional.*


*  *

- Would be very useful to write the excitation wavelength of the probes in the figure legends as well as emission bands.

***Authors’ reply:*** *As indicated in the methods section, bi-photon excitation was set at 720 nm, and this was true for absolutely all the microscopy experiments described in this manuscript. It therefore does not seem useful to specify this in all the legends as well.*

  

- Also, it would be extremely useful to have the figures a bit more informative and less dependent to the legend. In the current form, I had to go back and forth between the figure and the legend many times to understand the figure. Instead, if the authors put a bit of descriptive text on the figures, it would have been a lot easier to understand the figures (like they did for Figure 7 where they 3min, 15 min and 30 min as well as 4 uM and 200 nm on the figure itself). For instance, for figure 2, I would write; Laurdan, M-Laurdan, C-Laurdan on the rows and “brightfield”, “probe”, “lysotracker”, “merge” and “close-up” for the columns. Similarly I would do it for every figure so as soon as the reader looks at the figure, she can get what the figure tells without reading the legend. Similarly, they should add labels to Figure 5 and 8 on the figure what thin and bold lines or colors etc mean.

***Authors’ reply:*** *Thys type of presentation was used out of habit (many journals actually discourage the use of lettering and legends within the figures). But we agree with Dr. Sezgin that it would probably be better to have some legends within the figures, and have re-introduced them in the updated version of the manuscript.*


*  *

- Authors should comment on possible FRET between Laurdan and Nile Red when they do the spectral decomposition between these two.

***Authors’ reply:*** *What sort of comment? As underlined above, the analysis of the double staining experiment with laurdan and Nile Red is purely qualitative. We fail to grasp how FRET, if it actually occurred, would influence these qualitative results, and our conclusions.*


*  *

- Authors mention the “autoquenching” because of the decrease in signal with concentration. Are the authors sure that this is what happens? Can that be something else?

***Authors’ reply:*** *If we have not mentioned anything else, it is because we cannot think of any other mechanism that may realistically cause this effect. But we are open to suggestions …*

  

- 3min pictures in Fig7 is different than the rest, why?

***Authors’ reply:*** *At three minutes, the fluorescence intensities are much weaker because the probe had not had time to stain the cells completely. The signal to noise ratios were thus much lower, which explains the duller aspect of the pictures, both at 4uM and 200 nM. This has now been clarified in the figure legend.*

  

- For Figure 10-13, it is necessary to show some quantification, qualitative assessment with the colors isn’t enough.

***Authors’ reply:*** *We beg to differ on this subject. For a variety of reasons developed in the discussion, we feel that our results can only be considered as semi-quantitative. It is for this reason that we have chosen to represent the data with a LUT based on six discrete colors, and have refrained from carrying out any further quantitation. *

- “Considering the importance of temperature in regulating the state and order in lipid bilayers….” Authors should give a reference here such as Burns *et al.*, 2017 ^7^.

***Authors’ reply:*** *We thank Dr Sezgin for his suggestion and have included this reference in the updated version of the manuscript.*


*  *

- In the first paragraph of the Discussion, authors should also discuss the recently published improved approach of spectral imaging analysis (Aron *et al.*, 2017 ^8^)

***Authors’ reply:*** *Despite its title, this particular paper is not really about spectral imaging analysis, but about a bioinformatics toolbox for high throughput GP analyses. We do not see how this is relevant to our results.*

- In the discussion, when authors discuss the differences of lipid packing, they only consider the lipid composition. They should also add a short discussion on the presence of actin cytoskeleton for plasma membrane (and absence of it for intracellular membranes) may be responsible for relatively more ordered plasma membrane compared to intracellular ones (Dinic *et al.*, 2013 ^9^).

***Authors’ reply:*** *We thank Dr Sezgin for his suggestion and have included this reference in the updated version of the manuscript.*

**
